# Machine learning approaches for EGFR mutation status prediction in NSCLC: an updated systematic review

**DOI:** 10.3389/fonc.2025.1576461

**Published:** 2025-07-10

**Authors:** Liu Haixian, Pang Shu, Li Zhao, Lu Chunfeng, Li Lun

**Affiliations:** ^1^ Respiratory and Critical Care Medicine Center, Weifang People’s Hospital, Weifang, China; ^2^ The First Affiliated Hospital, Shandong Second Medical University, Weifang, China; ^3^ Precision Pathology Diagnosis Center, Weifang People’s Hospital, Weifang, China; ^4^ Critical care medicine, Weifang People’s Hospital, Weifang, China; ^5^ College of Mechanical Engineering and Automation, Weifang University, Weifang, China

**Keywords:** artificial intelligence, Non-small cell lung cancer (NSCLC), EGFR mutation, deep learning, medical imaging

## Abstract

**Background:**

With the rapid advances in artificial intelligence—particularly convolutional neural networks—researchers now exploit CT, PET/CT and other imaging modalities to predict epidermal growth factor receptor (EGFR) mutation status in non-small-cell lung cancer (NSCLC) non-invasively, rapidly and repeatably. End-to-end deep-learning models simultaneously perform feature extraction and classification, capturing not only traditional radiomic signatures such as tumour density and texture but also peri-tumoural micro-environmental cues, thereby offering a higher theoretical performance ceiling than hand-crafted radiomics coupled with classical machine learning. Nevertheless, the need for large, well-annotated datasets, the domain shifts introduced by heterogeneous scanning protocols and preprocessing pipelines, and the “black-box” nature of neural networks all hinder clinical adoption. To address fragmented evidence and scarce external validation, we conducted a systematic review to appraise the true performance of deep-learning and radiomics models for EGFR prediction and to identify barriers to clinical translation, thereby establishing a baseline for forthcoming multicentre prospective studies.

**Methods:**

Following PRISMA 2020, we searched PubMed, Web of Science and IEEE Xplore for studies published between 2018 and 2024. Fifty-nine original articles met the inclusion criteria. QUADAS-2 was applied to the eight studies that developed models using real-world clinical data, and details of external validation strategies and performance metrics were extracted systematically.

**Results:**

The pooled internal area under the curve (AUC) was 0.78 for radiomics–machine-learning models and 0.84 for deep-learning models. Only 17 studies (29%) reported independent external validation, where the mean AUC fell to 0.77, indicating a marked domain-shift effect. QUADAS-2 showed that 31% of studies had high risk of bias in at least one domain, most frequently in Index Test and Patient Selection.

**Conclusion:**

Although deep-learning models achieved the best internal performance, their reliance on single-centre data, the paucity of external validation and limited code availability preclude their use as stand-alone clinical decision tools. Future work should involve multicentre prospective designs, federated learning, decision-curve analysis and open sharing of models and data to verify generalisability and facilitate clinical integration.

## 1 Introduction

Non-small cell lung cancer (NSCLC), the most prevalent subtype of lung cancer, accounts for approximately 85% of all lung cancer cases (1). Within NSCLC, adenocarcinoma and squamous cell carcinoma represent the two most common histopathological subtypes. With the evolution of personalized and precision medicine, the detection of specific genetic mutations in NSCLC has become pivotal for stratifying patients based on therapeutic responsiveness. Among these, epidermal growth factor receptor (EGFR) mutation profiling is particularly critical, as EGFR a cell-surface receptor driving cellular proliferation and survival exhibits mutations that enhance sensitivity to tyrosine kinase inhibitors (TKIs). Clinically, EGFR-mutant patients are frequently characterized by non-smoking status, adenocarcinoma histology, female sex, and East Asian ethnicity (2–4).

In clinical practice, histopathological biopsy remains the gold standard for procuring tissue specimens and conducting mutational analysis to guide treatment planning. However, obtaining sufficient biopsy material for molecular profiling is not always feasible, particularly in high-risk patients with coagulopathies or comorbidities contraindicating invasive procedures. Furthermore, biopsy-derived tumor cells may inadequately capture intratumoral heterogeneity, reflecting only a limited spatial sampling of the tumor’s genomic landscape (5, 6). For instance, Taniguchi et al. (7) demonstrated that among 50–60 tumor regions analyzed in 21 EGFR-mutant patients, 28.6% exhibited intratumoral heterogeneity harboring both EGFR-mutated and wild-type subclones. Given that all patients with pulmonary masses undergo pre-treatment computed tomography (CT), these images serve as a rich data source for supplementary genomic interrogation, potentially identifying EGFR mutation carriers. Discordant findings between biopsy and CT-based analyses may warrant tumor re-sampling, thereby reducing the likelihood of missing actionable EGFR mutations. Consequently, quantitative characterization of CT-derived features has emerged as a critical adjunct for refining EGFR mutation status assessment.

Medical imaging has emerged as a pivotal platform for discovering and applying biomarkers in lung cancer. Recent investigations have employed artificial‐intelligence algorithms to quantify the biological, phenotypic and functional information embedded in imaging data, with radiomics and deep-learning approaches representing the two most prominent paradigms (8, 9). Radiomics relies on manual lesion delineation followed by extraction of high-dimensional texture and morphological features, enabling rapid screening and preliminary subtyping, yet it remains constrained in characterising tumour margins and the peritumoral micro-environment. By contrast, deep learning employs end-to-end networks that automatically learn image features without precise segmentation, capturing latent cues strongly associated with key outcomes—such as EGFR mutation status—while reducing labour costs. Moreover, deep networks can identify intratumoural subregions linked to genetic heterogeneity, thereby providing targets for image-guided biopsy. Each method offers distinct advantages, and both have demonstrated clinical potential in supporting diagnosis, response assessment and therapeutic decision-making, thus furnishing new imaging-based pillars for precision oncology in lung cancer (10–13).

This review seeks to advance understanding of AI applications in oncology by categorising current algorithmic strategies and summarising recent advances in predicting EGFR mutation status and subtypes in lung cancer.

## 2 Deep learning-based artificial intelligence technologies

### 2.1 Applications of deep learning in lung cancer diagnosis

**Figure 1:**
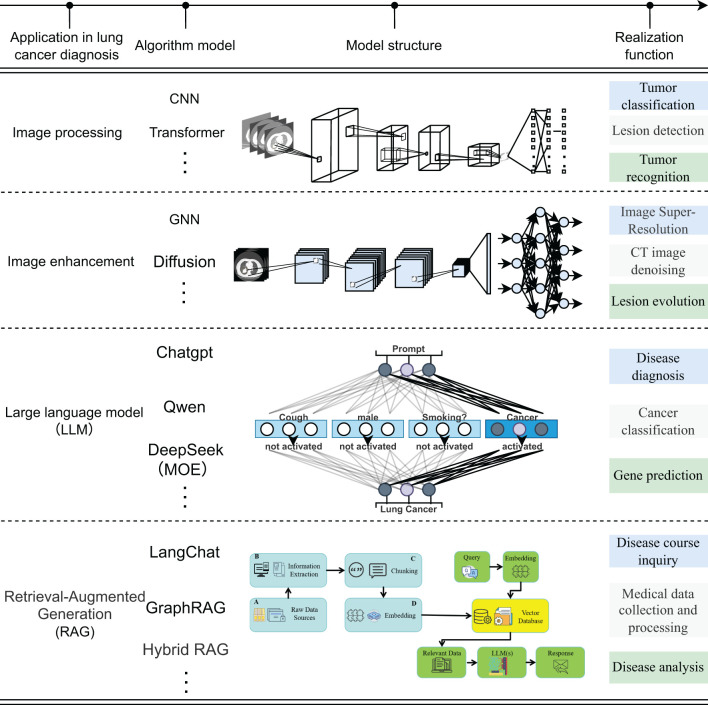
Application of deep learning technology in the diagnosis and treatment of lung cancer.

The current application of deep learning technology in the field of artificial intelligence for lung cancer images can be summarized into four aspects: ① Image processing: Convolutional neural networks focus on capturing local texture and morphology, while the Transformer relies on the self-attention mechanism to achieve cross-scale global feature integration; ② Image enhancement: Graph neural networks improve the contrast and consistency of CT/MR Images by modeling the topological relationship between pixels, and diffusion models significantly improve the resolution in low-dose or low-signal-to-noise scenes through iterative denoising. ③ Large language model: With the ability of deep semantic reasoning and context modeling, it integrates radiological images, pathological images and clinical texts to generate interpretable comprehensive diagnosis and genetic variation prediction reports; ④ Retrieval enhanced generation model: By integrating information retrieval and generation mechanisms, it enables the rapid aggregation of multimodal case knowledge and longitudinal disease course analysis, as shown in **Figure 1**. The four types of methods jointly demonstrate the potential of deep learning in automatic feature learning, multimodal data fusion, and intelligent decision support, and also highlight the ongoing challenges in terms of computing resources, data quality, and model interpretability (14–28).

### 2.2 Generalized framework for AI-driven prediction of genetic mutations in non-small cell lung cancer

**Figure 2:**
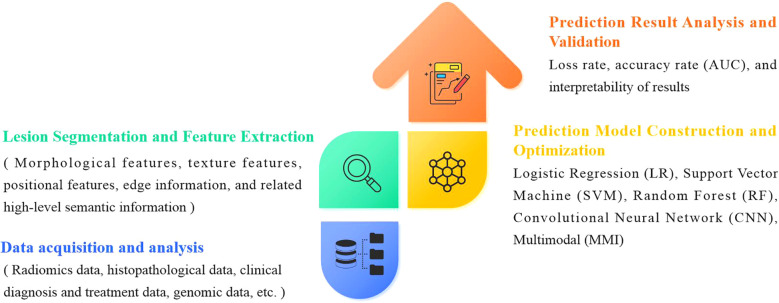
General framework for AI techniques to predict gene mutations in non-small cell lung cancer.

The evolution of artificial intelligence (AI) has necessitated the integration of multimodal data to improve the accuracy and reliability of predicting genetic mutations in non-small cell lung cancer (NSCLC). A standardized analytical workflow typically comprises four critical phases: (a) data acquisition and preprocessing, (b) lesion segmentation and radiomic feature extraction, (c) predictive model development and training optimization, and (d) prediction validation and clinical interpretation (29, 30), as illustrated in **Figure 2**.

During the data collection phase, case selection is restricted to pathologically confirmed lung adenocarcinoma patients with EGFR mutations (exon 19 deletions or L858R substitutions) verified by molecular testing. Corresponding radiological (CT) and histopathological imaging data are acquired concurrently. Exclusion criteria include: (1) prior antitumor therapy before CT imaging or EGFR genotyping, and (2) temporal misalignment between imaging, biopsy, and molecular testing procedures.

**Figure 3:**
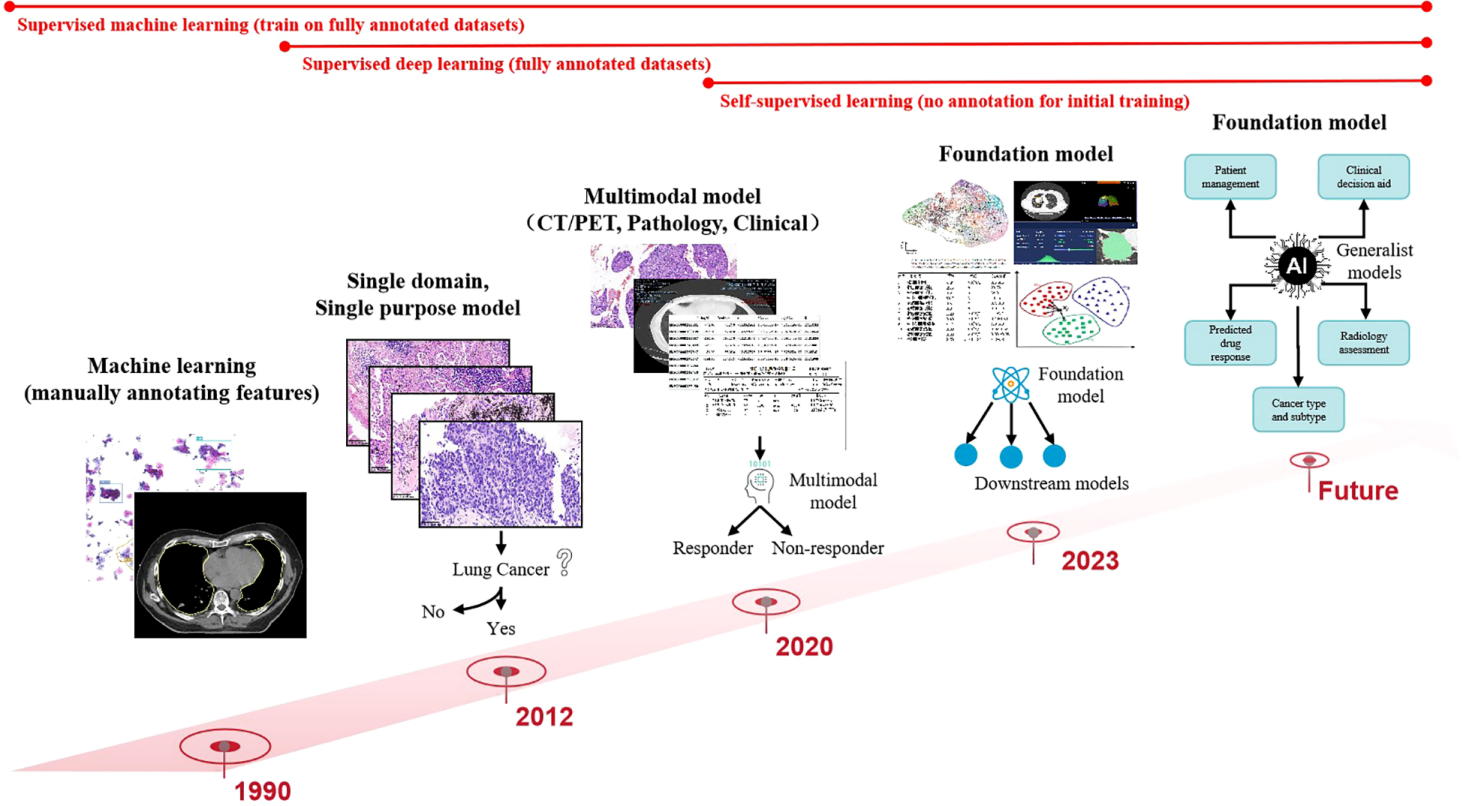
Computer vision has evolved from simple, specialized, shallow models to deep, multimodal, general-purpose models.

Data preprocessing forms the foundation for ensuring analytical validity in predictive modeling. In the early 2000s, AI applications in medical imaging predominantly employed supervised learning models relying on manually engineered features. These required domain experts to annotate imaging characteristics (e.g., tumor texture, margins) and construct fully labeled datasets for model training. Circa 2012, deep learning paradigms particularly convolutional neural networks (CNNs) revolutionized the field by autonomously learning hierarchical features directly from raw imaging data through end-to-end training on large annotated datasets, significantly enhancing model performance and generalizability. Post-2020, self-supervised learning emerged as a transformative approach, enabling feature extraction from unlabeled data without external annotations (31), as shown in **Figure 3**.

While manual annotation ensures high interpretability and accuracy, its labor-intensive nature limits scalability, making it suitable only for small, homogeneous datasets. In contrast, contemporary multimodal predictive models integrate radiomics (CT/PET), histopathology (whole-slide imaging), clinical metadata (e.g., ECOG status), and genomic profiles (e.g., EGFR variant allele frequency), achieving superior predictive accuracy for NSCLC mutations. Future advancements aim to develop large-scale, self-supervised foundation models pretrained on cross-modal unlabeled datasets. These models could be efficiently fine-tuned for diverse downstream tasks (e.g., mutation prediction, treatment response) with minimal task-specific training data. The ultimate objective is to create multifunctional AI systems capable of analytical reasoning, clinical interpretation, predictive modeling, and interactive decision support for patients and clinicians (32).

(1) Manual Tumor Feature Extraction

In the 1990s, early applications of machine learning for pulmonary tumor characterization predominantly relied on manual annotation by experienced radiologists or pathologists to delineate tumor regions. Radiomics-driven approaches enabled precise extraction of diverse tumor features, including morphological characteristics (e.g., size, shape, margin spiculation), textural patterns (e.g., gray-level distribution, heterogeneity), and locational attributes (e.g., lobar positioning). Post-extraction, feature selection techniques such as correlation coefficient analysis, least absolute shrinkage and selection operator (LASSO) regression, and principal component analysis (PCA) were applied to identify features most predictive of clinical endpoints. Selected feature sets were then input into machine learning models including logistic regression (LR), support vector machines (SVMs), and random forests (RFs) for malignancy grading, therapeutic response prediction, or prognostic stratification. Manual annotation leverages clinical expertise to capture nuanced visual patterns, particularly in cases with ill-defined tumor boundaries or irregular morphology, often outperforming automated methods in accuracy. Furthermore, manually derived features offer high interpretability for clinical decision-making. However, this approach suffers from inherent limitations: inter-observer variability, time-intensive workflows, and poor scalability to large datasets, rendering it impractical for modern precision medicine demands (33–35).

(2) Deep Learning-Based Feature Extraction

Deep learning automates tumor feature extraction through artificial neural networks (e.g., convolutional neural networks, CNNs) trained to hierarchically learn imaging signatures from radiomic data. These models capture both low-level textural/edge features and high-level semantic patterns (e.g., tumor shape, intralesional heterogeneity, peritumoral tissue interactions). Extracted features, represented as high-dimensional vectors, facilitate tasks such as tumor classification (benign vs. malignant), staging, treatment efficacy evaluation, and survival analysis. Dimensionality reduction techniques (e.g., PCA, t-distributed stochastic neighbor embedding [t-SNE]) may visualize these features before downstream model integration (36).

Deep learning offers advantages in processing large-scale data with minimal human intervention, ensuring feature consistency and efficiency. It excels at identifying subvisual patterns imperceptible to human observers, enhancing predictive performance. Nevertheless, critical challenges persist: the “black-box” nature of deep neural networks limits feature interpretability and clinical translatability; high-quality annotated datasets costly to acquire in medical domains are required for robust training; and overfitting risks escalate with limited data. Thus, while deep learning demonstrates transformative potential, its clinical adoption necessitates rigorous validation against domain knowledge and multimodal integration (37, 38).

## 3 Data sources and literature search strategy

This review was prepared following the Preferred Reporting Items for Systematic Reviews and Meta-Analyses (PRISMA 2020) guidelines and includes a systematic literature search with quality assessment, as well as an integrated evaluation of study quality and risk of bias. We provide a comprehensive analysis of recent artificial intelligence–based approaches for predicting epidermal growth factor receptor (EGFR) mutation status in non-small cell lung cancer (NSCLC), detailing the research methods, data modalities, and model performance (39, 40).

### 3.1 Search strategy

A systematic literature search was conducted up to December 2024 in PubMed (Medline), Web of Science, and IEEE Xplore. The search strategy combined controlled vocabulary (e.g., MeSH) and free‐text terms, including “EGFR,” “epidermal growth factor receptor,” “mutation,” “deep learning,” “artificial intelligence,” “radiomics,” “machine learning,” and “non‐small cell lung cancer,” with Boolean operators. To ensure comprehensiveness, we also performed supplementary screening by examining the reference lists of all relevant articles.

### 3.2 Inclusion and exclusion criteria

Study selection was performed according to the following inclusion and exclusion criteria: as shown in **Table 1**.

**Table 1:** Inclusion and exclusion criteria.

Inclusion criteria	Exclusion criteria
• Patients diagnosed with non-small cell lung cancer (NSCLC)	• Review articles, conference abstracts, editorials, or letters
• Studies employing AI techniques to predict EGFR mutation status;	• Non-human studies (e.g., animal or cell experiments)
• Original research (prospective or retrospective);	• Studies not involving EGFR prediction or without any AI–based analysis
• Reporting at least one model performance metric (e.g., accuracy, AUC, sensitivity, or specificity);	• Duplicate publications (only the most complete report retained)
• Full text available in English or Chinese	• Insufficient data to support quantitative analysis

### 3.3 Study selection process

**Figure 4:**
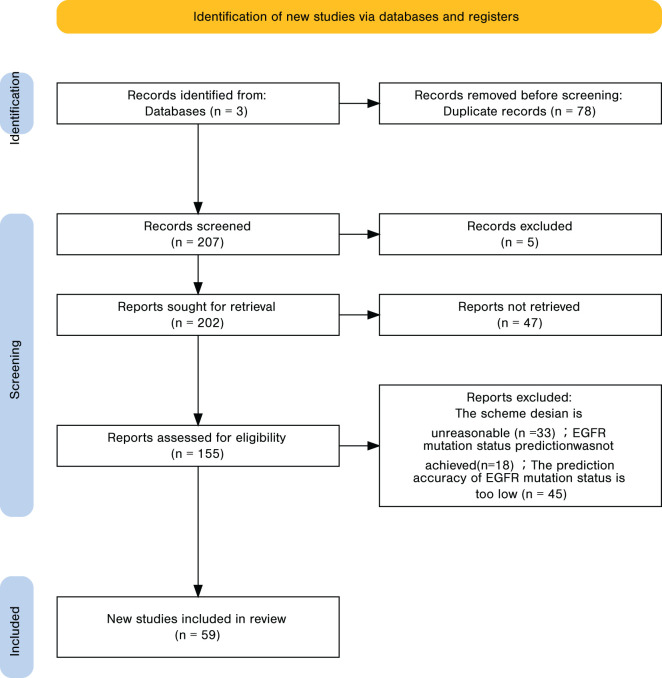
Literature data retrieval and screening process.

A total of 285 records were identified, of which 78 duplicates were removed, leaving 207 unique records. Title and abstract screening excluded 55 irrelevant studies, resulting in 155 articles for full-text review. Applying the predefined inclusion and exclusion criteria led to the exclusion of 96 articles, and 59 studies were ultimately included in the analysis. The detailed screening workflow is presented in **Figure 4**. For every included study, we recorded the point estimate of the AUC; when a 95% confidence interval (CI) was provided, it was extracted alongside the point estimate. If the CI was absent, the entry was labelled “NR (Not Reported)”, as noted in the footnote of the results table.

### 3.4 Risk of bias and applicability assessment

To systematically evaluate the methodological quality, risk of bias, and clinical applicability of the included studies, we applied the QUADAS-2 tool (Quality Assessment of Diagnostic Accuracy Studies 2). Eight original studies that developed AI models for EGFR mutation prediction using real-world clinical data were selected for this analysis.

QUADAS-2 evaluates:

1. Risk of Bias, across four domains:
  - Patient Selection
  - Index Test (i.e., the AI model)
  - Reference Standard (EGFR mutation detection method)
  - Flow and Timing (appropriateness of the sequence and interval between data collection and model evaluation)
2. Applicability Concerns, addressing the first three domains (Patient Selection, Index Test, Reference Standard) in terms of clinical relevance and generalizability.

**Figure 5:**
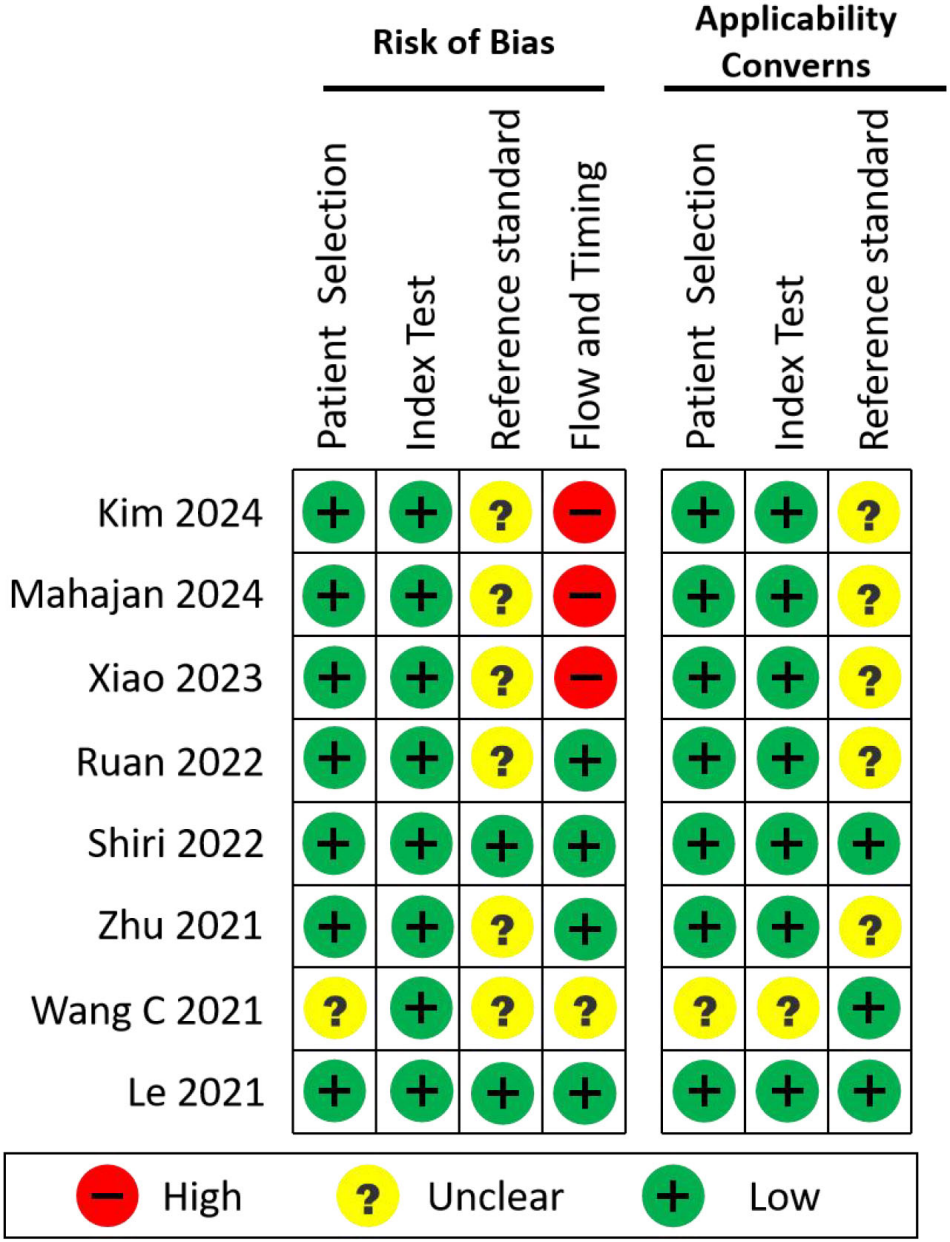
Assessment results (risk of bias and applicability assessment).

Two reviewers performed all assessments independently, and any discrepancies were resolved through discussion. The summary of these evaluations is presented in **Figure 5**.

## 4 Radiomics-based prediction of genetic mutation status in non-small cell lung cancer

**Figure 6:**
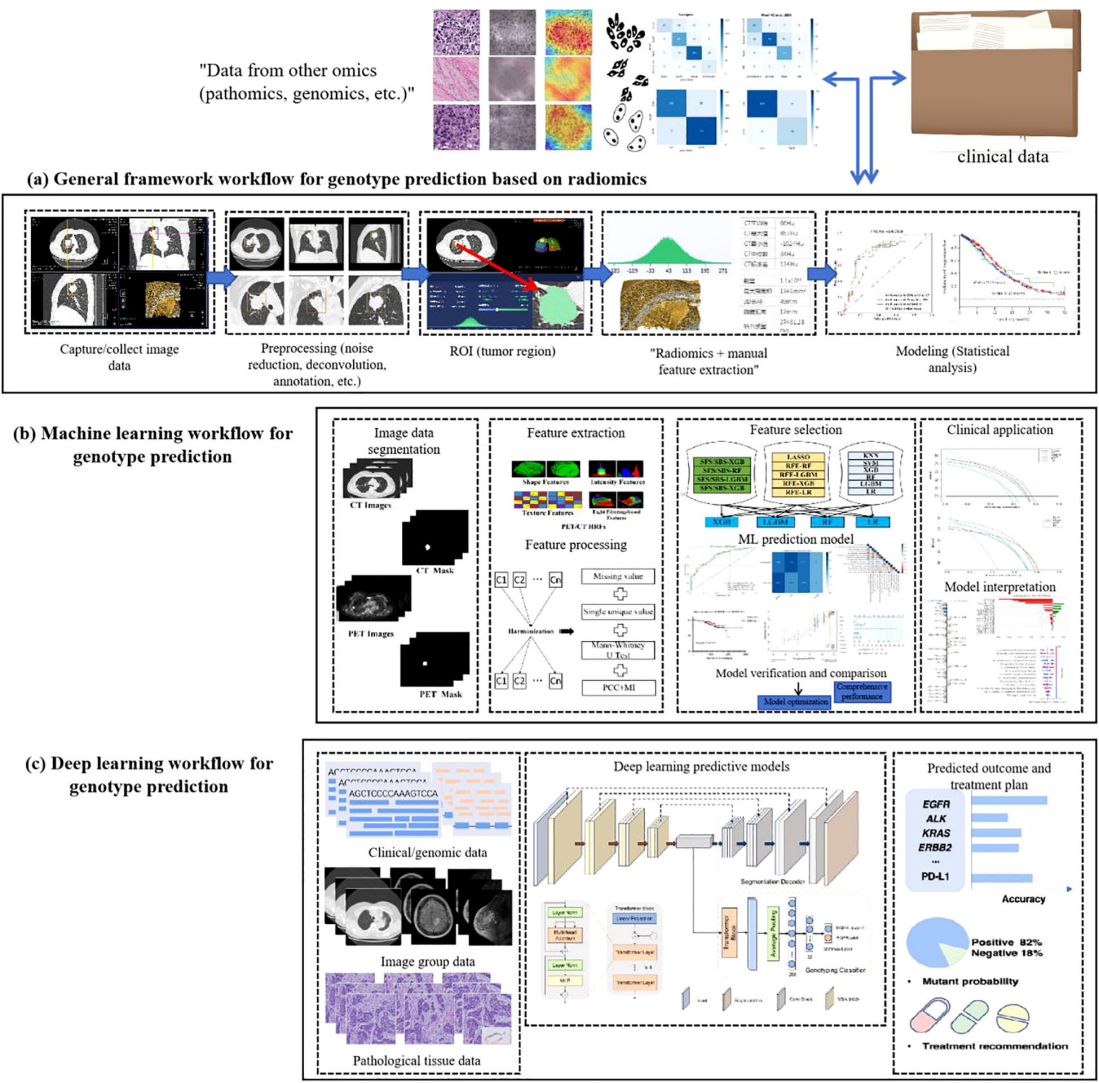
The processing of the three methods.

Radiomics is a systematic methodology encompassing the entire workflow from image acquisition to predictive performance evaluation. This approach involves critical steps: (1) image acquisition and reconstruction, (2) tumor segmentation, (3) feature extraction and filtering, (4) predictive model development, and (5) validation and performance assessment. During model construction, researchers commonly employ diverse classifiers for data analysis. Radiomics has evolved through three distinct phases based on classifier technologies: Traditional Statistical Radiomics (TSR), Machine Learning-based Radiomics (MLR) (particularly shallow learning algorithms), and Deep Learning (DL). These subcategories reflect iterative advancements in radiomics, each with unique characteristics and applicable clinical scenarios (**Figure 6**). TSR relies on statistical hypothesis testing (e.g., t-tests, ANOVA) to identify imaging biomarkers, while MLR leverages classical algorithms (e.g., SVM, RF) to map radiomic features to mutation probabilities. DL further automates feature engineering through hierarchical representation learning. Methodological divergences in data processing and feature analysis underscore both technological progress and context-specific problem-solving strategies (41, 42).

Traditional Statistical Radiomics (TSR) (43, 44) employs radiomic feature extraction (e.g., shape-, histogram-, and texture-based features) combined with statistical methodologies such as the least absolute shrinkage and selection operator (LASSO) to identify key features with non-zero coefficients. These features are then weighted to compute a radiomics score (Rad-score) for each lesion, representing a linear combination of selected features. In TSR, classical logistic regression (LR) serves as the primary classifier for model construction. Renowned for its simplicity and interpretability, TSR remains a foundational and clinically transparent approach in radiomics, enabling quantitative lesion characterization to inform diagnostic and therapeutic decision-making.

Machine Learning-based Radiomics (MLR) (41, 45) represents a mature, mainstream methodology for building classification/prediction models following feature extraction and optimization. Commonly used classifiers include random forests (RF), support vector machines (SVM), decision trees (DT), Bayesian networks (BN), and k-nearest neighbors (KNN). Subsequent to MLR, deep learning radiomics (DLR) (42) has emerged, leveraging artificial neural networks (ANNs) such as convolutional neural networks (CNNs) to extract deep features and construct predictive models. Unlike TSR and MLR which rely on manual feature engineering DL-based approaches utilize multi-layered nonlinear neural networks to automate feature learning directly from images via end-to-end workflows, eliminating human intervention.

### 4.1 Statistical prediction methods

Prior to the advent of radiomics, the prediction of EGFR mutation status in lung adenocarcinoma predominantly relied on clinical characteristics. Multiple studies have demonstrated significant associations between EGFR mutations and female sex, non-smoking status, and specific adenocarcinoma histologic subtypes. Additionally, CT imaging features including tumor maximal diameter, location, density, ground-glass opacity, pleural retraction, and air bronchogram have been validated as predictive biomarkers for EGFR mutations. Recent investigations further suggest that reduced tumor long-axis diameter correlates with increased EGFR mutation risk, while ground-glass opacity patterns are strongly linked to EGFR-mutant tumors. Yip et al. (43) highlighted the potential of radiomic features to quantify metabolic phenotypes for EGFR mutation prediction.

However, radiomic features primarily reflect imaging data and may insufficiently capture comprehensive disease profiles. To address this, researchers increasingly integrate clinical variables (including CT features) into radiomic models to enhance accuracy in identifying EGFR mutation status and subtypes. Recent studies indicate that models combining clinical and PET-derived features exhibit superior diagnostic performance and goodness-of-fit. For example, Li et al. (46) extracted 2,632 radiomic features from PET/CT images of 179 lung adenocarcinomas, randomly splitting the cohort into training (n=125) and testing (n=54) sets. Their models achieved AUCs of 0.708 and 0.652 for predicting exon 19 deletions and L858R mutations, respectively. Zhang et al. (45) retrospectively analyzed 18F-FDG PET/CT data from 173 NSCLC patients (71 EGFR+, 102 EGFR−), with 39% (68/173) at stages I/II and 61% (105/173) at stages III/IV. A combined PET/CT radiomics-clinical model achieved modest predictive performance (AUC=0.661). Nair et al. (47) demonstrated that PET/CT features outperformed CT alone in discriminating exon 19 and 21 mutations (AUC=0.86) using 326 features from 50 NSCLC patients, though limited sample size and absence of independent validation constrained generalizability.

Despite the rapid evolution of radiomics, traditional statistical regression (TSR) remains widely utilized due to its interpretability and clinical accessibility. TSR effectively transforms complex data into interpretable scores, often visualized via nomograms. However, TSR is limited by lower predictive efficiency compared to machine learning classifiers, driving the adoption of multivariate logistic regression (MLR) approaches.

### 4.2 Machine learning-based prediction methods

Machine learning (ML), a pivotal artificial intelligence (AI) methodology, involves constructing probabilistic statistical models from data to enable predictive analytics (48). Widely applied in medical imaging (MRI, CT, ultrasound), ML classifier selection requires careful consideration of data characteristics, model performance, computational resources, and clinical utility, with optimal algorithms typically identified through empirical validation.

Duan Yanan et al. (49) evaluated CT radiomics-driven ML models for EGFR mutation prediction in NSCLC. Their study enrolled 198 patients, extracting 1,050 radiomic features per case, ultimately selecting 16 features through dimensionality reduction. Seven classifiers logistic regression (LR), decision tree (DT), random forest (RF), neural network (NN), support vector machine (SVM), naïve Bayes (NB), and k-nearest neighbors (KNN) were tested. RF demonstrated superior performance, achieving AUC and F1 scores of 0.988/0.983 in the training cohort and 0.793/0.653 in the validation cohort. Delzell et al. (50) analyzed 416 quantitative imaging biomarkers from 200 lung nodule CT scans, employing three feature selection methods (linear combination filter, pairwise correlation filter, PCA) and three classifiers (linear, nonlinear, ensemble). Elastic net and SVM with linear/correlation-based feature selection yielded optimal tumor classification accuracy, while RF and bagged trees underperformed. Their findings underscore the efficacy of radiomic-ML integration in reducing false-positive rates. Naïve et al. (51) utilized gene expression (GDS3257) and DNA methylation β-values from The Cancer Genome Atlas (TCGA) to classify LUAD and LUSC subtypes. Bayesian and ReliefF/Limma feature selection identified 19 predictive genes, achieving AUC=0.89 across datasets. However, the absence of prospective validation limited clinical applicability. Key studies leveraging ML classifiers for NSCLC EGFR mutation prediction are summarized in **Table 2**, providing a comparative overview of recent advancements.

**Table 2:** Research on EGFR mutation prediction of lung cancer based on different machine learning classifiers.

Author/time of publication	Data	Training data set	Prediction model	Accuracy rate
Chang 2021 (52)	PET/CT	408	LR,nomogram, decision curve analysis	0.70~0.81
Guojin Zhang 2021 (53)	CT	546	DT,LR,SVM	0.74~0.4
Hong 2020 (34)	CT	140	NBS, KNN, RF, SVM, DT, LR	0.83–0.85
Jia 2019 (54)	CT	345	RF	0.65-0.83
Jiang 2019 (55)	PET/CT	80	LR, SVM	0.73-0.95
Jiang 2021 (56)	MRI	77	LR	0.63–0.77
Mu W 2020 (57)	PET/CT	175	LR	0.69–0.87
Le 2021 (58)	CT	143	RF, XGBoost	0.89
Liu G 2020 (59)	CT	210	LR	0.65–0.76
Lu L 2020 (60)	CT	105	KNN, SVM, RF, bagging	0.48–0.74
Ninomiya 2021 (61)	CT	99	SVM	0.65–0.86
Rossi 2021 (62)	CT	109	SVM	0.85
Shu Li 2019 (63)	CT	236	LR	0.57–0.81
Tu W 2019 (64)	CT	243	LR	0.53–0.82
Weng 2021 (65)	CT	210	LR	0.67–0.75
Wu 2020 (66)	CT	67	LR	0.84–0.97
Zhao 2019a (67)	CT	322	LR	0.73
Zhu 2021 (68)	CT	161	SVM	0.75–0.79
Ruan 2022 (69)	CT	100	SVM	0.793
Shiri 2022 (70)	CT	136	RF	0.92–0.94

Based on the summary analysis of **Table 2**, traditional machine learning (ML) demonstrates clear advantages, primarily due to the relative simplicity of the models, ease of implementation, and strong clinical interpretability. Commonly used algorithms, such as logistic regression (LR) and decision trees (DT), can explicitly highlight feature importance and decision pathways, making them particularly understandable and trustworthy for clinicians; consequently, they have been widely adopted in studies like those by Chang (2021), Wu (2020), Tu (2019), and Weng (2021). Furthermore, certain studies, including that by Duan et al., utilized random forest (RF) algorithms, achieving notably high training performance (AUC: 0.988, accuracy: 0.983) by effective feature selection (reducing 1050 features down to 16), indicating that traditional ML can provide excellent internal predictive performance under suitable data scales and optimized feature engineering.

However, significant limitations are also evident in traditional ML-based EGFR mutation prediction studies. First, the predictive performance of these models varies substantially among different studies, even when applying the same algorithm. For instance, the accuracy of LR reached as high as 0.97 in Wu (2020), whereas Lu (2020) reported accuracies ranging from only 0.48 to 0.74 using algorithms such as KNN, SVM, and RF. This highlights significant instability, potentially attributable to differences in imaging protocols, feature selection methodologies, and inherent dataset heterogeneity across studies. Second, most of these studies included relatively small sample sizes (generally between 100–300 cases), predominantly from single-center retrospective cohorts, which may not adequately represent broader population characteristics or variations in imaging protocols, thereby limiting generalizability. Additionally, very few studies (e.g., Duan et al.) have reported independent external validation results, where external validation performance (AUC: 0.793) was substantially lower compared to internal training results (AUC: 0.988), reflecting significant overfitting risks and limited cross-institution applicability.

Overall, although traditional ML models hold advantages in terms of interpretability and implementation costs, the insufficient generalizability, sensitivity to data quality and feature selection, and instability across diverse clinical settings remain critical issues requiring further attention. Thus, future research should prioritize increasing sample sizes and data diversity, optimizing feature selection strategies, enhancing model generalizability, and emphasizing multicenter and independent external validations to facilitate the broader clinical implementation and application of traditional ML-based models.

### 4.3 Deep learning-based prediction of EGFR Gene mutations

Deep learning-based approaches for analyzing lung tumors and predicting genetic mutations hold broad prospects and significant clinical value, offering critical implications for improving therapeutic outcomes and patient survival rates. Research on constructing classification and predictive models for lung cancer using deep learning techniques has emerged as a global research focus. Deep learning demonstrates substantial potential for automating feature extraction processes from medical images, thereby streamlining workflows and enhancing predictive analyses (71). By autonomously learning abstract, high-level features from datasets and continuously improving model performance through iterative training, deep learning has enabled researchers to develop models for predicting EGFR mutation status with promising results. These advancements bridge fundamental, translational, and clinical research in non-small cell lung cancer (NSCLC).

Xiao et al. (72) collected PET/CT imaging data from 150 EGFR-mutant patients between 2016 and 2019, generating 3,794 PET/CT fusion datasets after 2D slicing (1,913 wild-type and 1,881 EGFR-mutant samples). Their study proposed a deep learning framework based on the EfficientNet-V2 model. First, 32 two-dimensional views were extracted from 3D cubic volumes of each pulmonary nodule. Deep features from these views were then utilized to predict EGFR mutation status. The deep learning model achieved AUCs of 83.64% and 82.41%, respectively, demonstrating promising efficacy in EGFR mutation prediction.

Seonhwa Kim et al. (73) retrospectively analyzed CT scans and clinical data from 1,280 NSCLC patients tested for EGFR mutations (454 mutant-type and 826 wild-type). The team developed a novel hybrid method integrating deep learning and radiomics to predict EGFR mutations. Radiomic features were extracted from preprocessed CT images of NSCLC tumors and combined with tumor images and clinical data as input for the predictive model. This approach achieved AUCs of approximately 0.81 and 0.78 in the initial cohort and external validation, respectively, highlighting the feasibility of combining radiomic analysis with deep learning for EGFR mutation prediction.

Chengdi Wang et al. (74) collected clinical information, histopathology reports, CT imaging data, and genetic testing results from 1,262 patients. The dataset was partitioned into training (N=882), validation (N=125), and test (N=255) sets at a 7:1:2 ratio. They proposed a novel deep learning method to predict EGFR mutation and PD-L1 expression status in NSCLC patients, integrating selected features to construct a prognostic model. A 3D convolutional neural network (CNN) was employed, achieving AUCs of 0.96 (95% CI: 0.94–0.98), 0.80 (95% CI: 0.72–0.88), and 0.73 (95% CI: 0.63–0.83) in the training, validation, and test cohorts, respectively.

Abhishek Mahajan et al. (48) analyzed CT imaging data from 990 patients with primary lung adenocarcinoma confirmed by genetic testing. The team developed and validated a deep learning-based radiogenomics (DLR) model combined with radiomic features to predict EGFR mutations in NSCLC, while evaluating semantic and clinical features associated with mutation detection. An end-to-end pipeline was applied to CT images from two NSCLC trials without precise segmentation. Two 3D CNNs were used to segment lung masses and nodules. The combined radiomic-DLR model achieved an AUC of 0.88 ± 0.03 for EGFR mutation prediction, outperforming individual models. Integration of semantic features further improved accuracy, yielding an AUC of 0.88 ± 0.05.

Shuo Wang et al. (75) compiled CT imaging and EGFR sequencing data from 18,232 lung cancer patients across nine cohorts in China and the U.S., including a prospective Asian cohort (n=891) and The Cancer Imaging Archive (TCIA) cohorts of White populations stratified into thick-slice and thin-slice CT groups. The authors proposed a fully automated artificial intelligence system (FAIS) to extract whole-lung information from CT scans for predicting EGFR genotype and prognosis of EGFR-TKI therapy. FAIS was evaluated using AUC for EGFR genotype prediction and Kaplan-Meier analysis for progression-free survival (PFS) in EGFR-TKI-treated patients.

Wentao Zhu et al. (76) analyzed CT scans from 191 patients with biopsy-confirmed lung adenocarcinoma (LUAD) and squamous cell carcinoma (LUSC). They introduced a self-generating hybrid feature network (SGHF-Net) to classify lung cancer subtypes on CT images. A pathological feature synthesis module (PFSM) was innovatively designed to quantify cross-modal correlations via deep neural networks, deriving “gold-standard” pathological information from CT images. Simultaneously, a radiomic feature extraction module (RFEM) fused CT-derived features with pathological priors under an optimized framework, enhancing the model’s ability to generate discriminative and subtype-specific features for accurate prediction.

Le NQK et al. (77–79) compiled CT imaging and bed data from 576 patients diagnosed with non-small cell lung cancer (NSCLC). The dataset was partitioned into a training set (N = 420) and a test set (N = 156). A multimodal deep learning framework was subsequently developed, integrating 3D CNN survival analysis and DeepSurv methodologies. By combining deep radiomics, traditional radiomics, and clinical parameters, the model predicted the survival status of NSCLC patients. The findings indicate that the DeepSurv CT deep radiomics model outperforms the conventional Cox-PH model, and the integration of multiple parameters enhances prediction accuracy.

Other research findings regarding the prediction of EGFR mutations in lung cancer using deep learning methods, along with detailed information on data sources, scanner heterogeneity, and validation designs, are presented in **Table 3**.

**Table 3:** Studies on prediction of EGFR mutation in lung cancer based on deep learning methods.

Author/time of publication	Data	Training set	Method	Type of validation	Aceuraey rate	Multicenter	External verification	Cross-vendor scanning
Shuo Wang 2019 (80)	CT	844	DL	5-fold cross validation and independent validation with two-center data	0.81 (95% CI:0.79 - 0.83)	Yes	Yes	Yes
Shuo Wang (81)	CT	18232	DL	Independent validation with multi-center data	0.748-08 13(95% CI:0.732 - 0.853)	Yes	Yes	Yes
Dongqi Gui 2022 (82)	CT	280	DL	Independent validation	0.8599 (NR)	Yes	Yes	Yes
Wei Mu,2020 (62)	PET/CT	616	DL	Independent validation with multiceter data	0.81 (NR)	Yes	Yes	Yes
Chengdi Wang, 2022 (83)	CT	1135	DL	5-fold cross validation with single hospital data	0,950 (95% CI, 0.938 - 0.960), 0.934, (95% CI, 0.906 - 0.964) and 0.946 (95% CI, 0.933 - 0.958) for PD-L1 expression signature <1%, 1~49%, and≥ 50%	No	No	Yes
Zhengbo Song, 2021 (84)	CT	937	DL	5-fold cross validation with three hospitals data	0.7754 (95% CI 0.7199–0.8310)	Yes	Yes	Yes
Panwen Tian, 2021 (85)	CT	939	DL	Independent validation with single-center data	0.76 (95% CI: 0.66~0.85)	No	No	No
Chengdi Wang, 2022 (86)	CT	818	DL	Independent validation with single-center data	0.842 (95% CI, 0.825-0.855)	No	No	No
Tiening Zhang, 2021 (87)	CT	134	ML DL	5-fold cross validation with single-center data	0.78 (95% CI: 0.70–0.86)	No	No	No
Zhao,Yu,2024 (88)	CT	1719	DL	15-fold cross validation with single-center data	0.82 (95% CI 0.71 ~ 0.93) ~ 0.96(95%0.91 ~ 1.00)	Yes	Yes	Yes
Wang S,2021 (80)	CT	844	DL	Independent validation with single-center data	0.81 (95% CI 0.79-0.83)	Yes	Yes	Yes
Song J,2021 (89)	CT	665	DL	Independent validation with single-center data	0.78 (NR)	Yes	Yes	Yes
ZhangX,2024 (90)	CT	508	DL	Independent validation with single-center data	0.884 (95% CI 0.876 - 0.892)	Yes	Yes	Yes
Dong Y,2021 (91)	CT	363	DL	Independent validation with multi-center data	0.7943 (NR)	Yes	Yes	Yes

In medical imaging, acquiring large-scale training datasets remains challenging. Transfer learning (TL) strategies theoretically address this limitation by leveraging visual feature similarities across domains (92). However, the scarcity of pre-trained models in medical imaging restricts TL applications. Recent advances propose semi-supervised learning techniques, such as pseudo-labeling and generative adversarial networks (GANs), to utilize unlabeled data and mitigate sample size constraints (93). These innovations hold significant potential for EGFR mutation prediction and warrant further exploration (94). Additionally, training deep learning models demands substantial computational resources (e.g., CPUs, GPUs, and memory). In resource-limited settings, traditional regression (TR) may offer a pragmatic alternative. The “black box” nature of deep neural networks also poses interpretability challenges, as their high complexity and multi-parametric architecture obscure internal decision-making processes (95).

Through comprehensive analysis, deep learning (DL) models exhibit remarkable advantages in the automatic extraction of medical imaging features and classification accuracy, with most studies reporting predictive accuracies (AUC values) ranging between 0.76 and 0.96. For instance, the DL-based approach proposed by Wang et al. demonstrated high stability across a multicenter independent validation dataset, achieving an AUC ranging from 0.748 to 0.813, indicating potential cross-institutional generalizability. Additionally, several studies (e.g., Gui, 2022; Zhang, 2024) highlight that deep learning techniques, particularly through advanced feature extraction and multimodal data integration (e.g., PET/CT combined with clinical information), significantly enhance prediction performance, reaching AUC values as high as 0.86 to 0.88.

However, current DL models for EGFR mutation prediction still encounter notable limitations and challenges. Firstly, most studies predominantly rely on single-center datasets (more than half conducted validations solely within single-center cohorts), with limited multicenter and cross-device validation, potentially restricting the model’s true generalizability. Specifically, among the published studies, only 17 out of 59 (29%) conducted external validation using independent datasets, and merely 10 out of 59 (17%) involved multicenter validations using scanners from different manufacturers. Notably, the pooled AUC was 0.86 ± 0.07 on internal test sets, yet this performance decreased to 0.77 ± 0.06 during external validations. Secondly, substantial variations in sample size exist among studies, ranging from several hundred to tens of thousands of cases (e.g., Wang et al. included a sample size of 18,232), highlighting the lack of consensus on data diversity and standardization across research efforts. Additionally, the inherent “black box” nature of DL models significantly limits their clinical interpretability.

In conclusion, future research should prioritize expanding multicenter validation datasets, standardizing imaging protocols and data quality control, and enhancing model interpretability studies, ultimately promoting the practical application and widespread adoption of deep learning models in clinical practice.

## 5 Novel artificial intelligence approaches for predicting EGFR mutations in non-small cell lung cancer

Although radiomics-based machine learning (ML) and deep learning (DL) models have demonstrated potential in EGFR mutation detection (e.g., CT radiomics models achieving AUCs of 0.82–0.88) (96, 97), current studies remain constrained by several limitations. First, single-modality imaging data primarily reflect tumor morphology or functional characteristics, failing to capture molecular dynamics or microenvironmental heterogeneity linked to EGFR mutations. Second, traditional models rely on manual feature engineering or isolated DL architectures, limiting their capacity to model cross-scale biological correlations and resulting in poor interpretability (only ~35% of key feature contributions are explainable). Third, most studies utilize static imaging data, lacking dynamic tracking of EGFR mutation evolution during treatment. To address these challenges, novel artificial intelligence (AI)-driven multimodal data fusion strategies are emerging as pivotal solutions. By integrating radiomics, liquid biopsy, pathomics, and dynamic clinical data, next-generation models can construct cross-dimensional feature association networks (e.g., spatiotemporal coupling of imaging texture features and ctDNA methylation profiles). Leveraging graph neural networks (GNNs) and federated learning, these models enable collaborative optimization of multicenter heterogeneous data, enhancing predictive performance (AUC projected to exceed 0.95) while elucidating EGFR mutation-driven mechanisms and drug resistance evolution, thereby supporting precision therapeutic decision-making across the treatment continuum (15, 98).

### 5.1 Multimodal data fusion for predicting lung cancer gene mutations

Recent advances in multimodal data fusion have significantly improved EGFR mutation prediction in non-small cell lung cancer (NSCLC). Researchers worldwide have developed innovative predictive models by integrating radiomics, genomics, pathology, and clinical data. The core advantage of multimodal models lies in their ability to fuse heterogeneous, multidimensional data for comprehensive and precise EGFR mutation prediction (99–102). The analytical workflow typically involves four key stages:

1. Data Preprocessing: Standardization of heterogeneous data sources, including CT image resampling and normalization, genomic variant annotation and feature selection, and clinical data imputation and encoding.
2. Feature Extraction: High-dimensional feature extraction from multimodal data using DL or traditional ML methods. Examples include texture feature extraction from CT images via convolutional neural networks (CNNs) and semantic feature extraction from pathology reports using natural language processing (NLP).
3. Feature Fusion: Alignment and integration of multimodal features through early fusion, late fusion, or intermediate fusion strategies. Common techniques include graph neural networks (GNNs), attention mechanisms, and multimodal Transformer architectures.
4. Model Training and Validation: Performance evaluation via cross-validation or independent test sets, coupled with interpretability tools (e.g., SHAP values or Gradient-weighted Class Activation Mapping [Grad-CAM]) to quantify key feature contributions.

This approach not only enhances EGFR mutation prediction accuracy (AUC improvements of 10%–15%) but also provides novel insights into the interplay between imaging features and molecular mechanisms.

Internationally, research consortia have demonstrated leading expertise in imaging-genomics joint modeling, multi-omics integration, and dynamic predictive system development. The Harvard Medical School team (2021) proposed the “Radiogenomic Fusion Network” (103), which employs graph convolutional networks (GCNs) to achieve deep integration of high-resolution CT imaging features with whole-exome sequencing (WES) data. Validated in a cohort of 412 NSCLC patients, the model achieved an AUC of 0.93 for EGFR mutation prediction and first identified a specific association between “ground-glass opacity” on CT imaging and the EGFR L858R mutation. The Memorial Sloan Kettering Cancer Center (MSK) Cancer Data Science Initiative Group (2024) (104) developed a framework integrating patient-reported clinical genomic data with natural language processing (NLP) techniques and multimodal biomarkers to improve the accuracy of overall survival (OS) prediction in cancer patients. The research team constructed a comprehensive dataset encompassing patients with non-small cell lung cancer (NSCLC), breast cancer, colorectal cancer, and other malignancies. NLP methodologies were employed to extract critical information from unstructured textual data, including clinical notes and diagnostic reports, while structured data—such as treatment histories, survival outcomes, and tumor characteristics—were systematically integrated to build a multimodal predictive model. Through rigorous cross-validation and external validation, the NLP component demonstrated stable performance across cancer subtypes. Notably, in NSCLC cohorts, the model achieved a precision of 0.78, an AUC of 0.98, and a recall of 0.92 for identifying prior treatment histories. The multimodal fusion approach effectively processed heterogeneous data types (unstructured text and structured clinical parameters), enabling robust survival prediction. This methodology provides clinicians with enhanced capabilities for personalized treatment planning, potentially improving therapeutic outcomes and patient survival rates.The research team led by Rui-Jiang Li and Sen Yang at Stanford University developed MUSK(2025) (105), a pre-trained foundational model based on the BeiT3 architecture. MUSK effectively leverages unlabeled and unpaired image-text data through a unified masked modeling approach. The model was trained on an extensive dataset comprising 50 million pathological image patches and 1 billion text tokens. To address the distinct visual characteristics and data distribution differences between pathological images and natural images, the team implemented several tailored optimizations: a multi-scale training strategy, pathological staining data augmentation, noisy data bootstrapping enhancement, and fine-grained multimodal alignment techniques. These methodological adaptations significantly improved the model’s learning capability for pathological data, resulting in enhanced clinical prediction accuracy.

Domestically, multimodal modeling research has prioritized clinical translation and optimization for region-specific healthcare contexts. The team led by Professor Lu Shun at Shanghai Chest Hospital, affiliated with Shanghai Jiao Tong University (2024) (106), innovatively integrated multiple non-invasive biomarkers to explore the potential for early identification of non-small cell lung cancer (NSCLC) patients who may derive durable clinical benefits from immune checkpoint inhibitor (ICI) therapy. The team developed a multiparameter predictive model incorporating standardized bTMB, dynamic changes in ctDNA during early treatment, and RECIST response to predict durable clinical benefit (DCB) from ICI therapy. This model demonstrated robust predictive performance in both the training and validation cohorts, with AUC values of 0.854 and 0.798, and accuracy rates of 79.5% and 74.7%, respectively. The inclusion of RECIST response further enhanced the model’s predictive capability, particularly in the validation cohort, where both sensitivity and specificity showed improvement.The research team led by Professor Li Weimin at Sichuan University (2024) (107) developed a multimodal artificial intelligence (MMI) system that integrates multidimensional clinical data—including clinical texts, imaging data, and laboratory indicators—to achieve accurate prediction of pulmonary infectious diseases, pathogen types, and timely identification of critical illness, thereby providing robust support for clinical decision-making. The MMI model was trained on 24,107 patient records comprising clinical texts and CT images to distinguish bacterial, fungal, viral pneumonia, and tuberculosis. The system demonstrated exceptional performance in both internal and external validation datasets, achieving AUC values of 0.910 (95% CI: 0.904–0.916) and 0.887 (95% CI: 0.867–0.909), respectively, comparable to the diagnostic accuracy of experienced clinicians. Furthermore, the MMI system rapidly differentiated viral and bacterial subtypes, with mean AUCs of 0.822 (95% CI: 0.805–0.837) for viral subtypes and 0.803 (95% CI: 0.775–0.830) for bacterial subtypes. Notably, the system also facilitated personalized medication recommendations to mitigate antibiotic misuse and exhibited significant advantages in predicting critical illness risks, offering a promising tool to optimize clinical workflows.A research team led by Beihang University (2022) (95) developed a fully automated artificial intelligence system (FAIS) that leverages whole-lung CT imaging information to predict EGFR genotype and evaluate prognosis in patients receiving EGFR-TKIs therapy. This multicenter study encompassed 18,232 lung cancer patients across nine Chinese and American cohorts, incorporating both prospective and retrospective data from Asian and Caucasian populations. Participants were stratified into thick- and thin-section CT groups. FAIS demonstrated capability in predicting EGFR mutation status and progression-free survival for EGFR-TKIs treated patients, with performance validated through AUC metrics and Kaplan-Meier analysis. Compared with two tumor region-based deep learning models, FAIS showed superior performance across multiple test cohorts, achieving AUC values ranging from 0.748 to 0.813. The FAIS-C model integrating clinical factors exhibited significant correlation with EGFR-TKIs therapeutic outcomes (log-rank p < 0.05), suggesting its potential as an effective complement to genetic sequencing methods.

### 5.2 Applications of large language models

Large Language Models (LLMs) are deep learning-based natural language processing models trained on massive textual datasets, enabling comprehension and generation of human language. Their core architecture typically relies on the Transformer framework, which utilizes self-attention mechanisms to capture long-range dependencies in text, facilitating deep contextual semantic understanding. Recent advances in computational power and data scalability have demonstrated the robust capabilities of LLMs across diverse domains, exemplified by models such as the GPT (Generative Pre-trained Transformer) series, DeepSeek, Qwen series, BERT (Bidirectional Encoder Representations from Transformers), and ChatGLM. These models excel not only in general natural language tasks (e.g., text generation, translation, and question answering) but also show significant potential in medical applications (108, 109).

**Figure 7:**
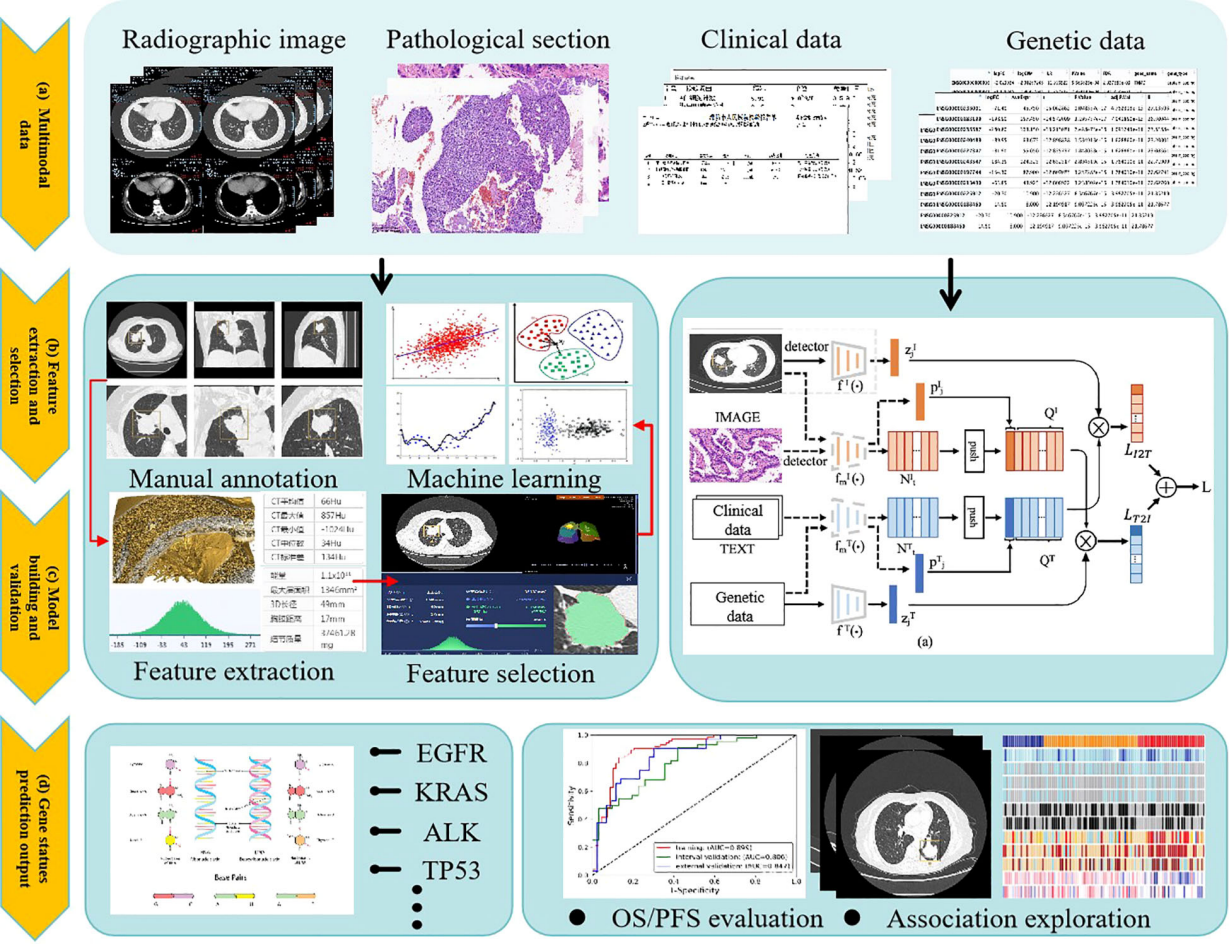
General framework for predicting gene mutation based on multimodal fusion method.

**Figure 8:**
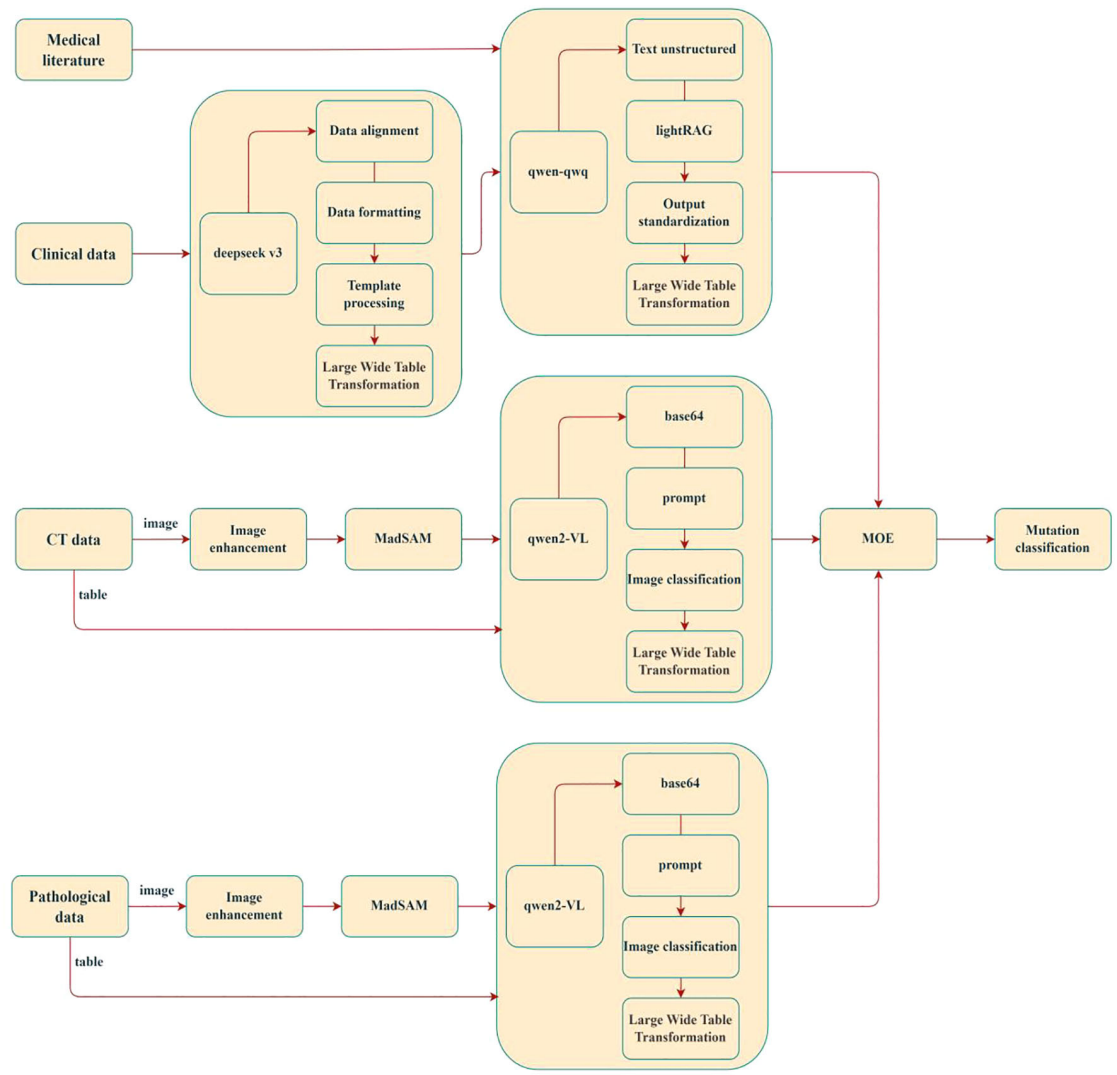
Classification process of NSCLC gene mutation predicted by LMOE model.

In recent years, the utility of LLMs in predicting epidermal growth factor receptor (EGFR) mutations in non-small cell lung cancer (NSCLC) has garnered substantial interest. Leveraging their advanced natural language processing and contextual reasoning capabilities, LLMs can effectively integrate textual information from multimodal data (e.g., pathology reports, clinical notes, and genomic annotations) to enhance predictive performance. Researchers have proposed novel technical approaches to optimize LLMs for lung cancer mutation prediction, achieving promising results (27, 110, 111). As illustrated in **Figure 7**, the LMOE (Large Mixture of Experts) framework exemplifies such innovation. This model integrates multimodal inputs—including medical imaging (CT scans, histopathology images) and clinical data—through image enhancement, data alignment, and structured processing to construct a unified high-dimensional feature space (wide-table transformation). It employs a lightRAG module for cross-modal semantic association and utilizes Qwen-series algorithms for final mutation classification. The LMOE framework directly addresses critical challenges in integrating multi-source heterogeneous clinical data, offering the following advantages:

1. Depth of Data Integration: Moving beyond traditional single-modality analyses, the model fuses histopathological texture features (via the MadSAM module), 3D spatial information from CT imaging, and temporal clinical parameters to better reflect the biological heterogeneity of lung cancer. For example, dynamic correlations between locally enhanced histopathological features (e.g., H&E-stained regions) and volumetric changes in CT-detected pulmonary nodules may reveal radiomic biomarkers specific to mutations such as EGFR or ALK.
2. Clinical Interpretability: A standardized output module maps predictions to mutation classification systems in clinical guidelines (e.g., NCCN criteria), ensuring model outputs directly inform targeted therapy decisions. This end-to-end clinical alignment significantly outperforms traditional “black-box” models.
3. Technical Extensibility: The Mixture of Experts (MOE) architecture supports phased validation, where clinical data serve as prior knowledge to constrain model training. Additionally, the Qwen2-VL module’s vision-language alignment capability provides scalable interfaces for integrating pathology report text with image features, as shown in **Figure 8**.

Multiple international and domestic research teams have conducted in-depth investigations into the application of large language models (LLMs) for predicting lung cancer gene mutations, achieving notable progress. For instance, the Google Health team (2022) (112) proposed the “Med-PaLM” model, which fine-tunes LLMs (e.g., GPT-3) to integrate pathology reports with radiomics data, achieving an AUC of 0.89(NR) in NSCLC EGFR mutation prediction, significantly outperforming conventional methods. The “BB-TEN” model, developed by a Columbia University research team (2025) (113), enables automated TNM (tumor size, regional lymph node involvement, and distant metastasis) classification from pathology report text. This framework employs a BERT architecture for semantic analysis of pathology reports combined with CT imaging features. Evaluated on nearly 8,000 pathology reports from Columbia University Medical Center, the model demonstrated robust performance with AUC values ranging from 0.815 to 0.942(NR). Harvard Medical School(2024) has developed PathChat (114), a vision-language general-purpose AI assistant for human pathology slide analysis. The system was pre-trained via self-supervised learning on image patches derived from over 1 million histopathological slides, enabling accurate disease identification from biopsy specimens with an accuracy rate approaching 90%. This performance surpasses that of GPT-4V, demonstrating its advanced diagnostic capabilities in computational pathology. In the same year, Professor Kunxing Yu at Harvard University developed the Clinical Histopathology Imaging Evaluation Foundation (CHIEF) model (115), a foundational framework for histopathological image analysis. The CHIEF model demonstrates diagnostic capabilities for 19 cancer types originating from pulmonary, breast, prostate, colorectal, gastric, esophageal, renal, cerebral, hepatic, thyroid, pancreatic, cervical, uterine, ovarian, testicular, cutaneous, soft tissue, adrenal, and bladder tissues, achieving a diagnostic accuracy approaching 94%. The research team led by Guangyu Wang at Beijing University of Posts and Telecommunications has developed “MedFound,” (116) a 176-billion-parameter medical large language model (LLM). This general-purpose model was pre-trained on a large-scale corpus comprising diverse medical texts and real-world clinical records. Through fine-tuning, MedFound employs a self-bootstrapping chain-of-thought approach to emulate physicians’ diagnostic reasoning processes, while incorporating a unified preference alignment framework to ensure consistency with standardized clinical practices. A study conducted by the First Affiliated Hospital of Sun Yat-sen University on the imaging and pathological evaluation of thyroid nodules demonstrated that in a comparison of 1,161 thyroid nodule imaging diagnoses from 725 patients, ChatGPT 4.0 and Bard exhibited significant to nearly perfect internal consistency. This performance was comparable to the human-machine interaction strategies employed by two senior radiologists and one junior radiologist, and surpassed the strategy involving only a single junior radiologist (117). Additionally, a large diagnostic model for pneumoconiosis, named “PneumoLLM” (118), developed by Chinese researchers, has established a novel paradigm for applying Large Language Models (LLMs) to data-scarce occupational diseases, as shown in **Table 4**. Extensive experiments have demonstrated the superior diagnostic capabilities of this large model in identifying pneumoconiosis.

**Table 4:** Multimodal predictive modeling in lung cancer research.

Author and time of publication	Data source	Training set size	Method	Feature extraction	Prediction model	Accuracy/AUC
Harvard Medical School 2021 (103)	CT + WES	412 Cases	GCN	3D texture feature	Radiogenomic Fusion Network	0.93 (NR)
Memorial Sloan Kettering Cancer Center 2022 (104)	Clinical data + genome	24950 Cases	multimodal	Context information Genomic data	Multimodal prediction model	0.98 (NR)
Stanford University2023 (105)	Clinical data +pathological images	11577 Cases	multimodal	Pathological morphological characteristics	MUSK model	–
Shanghai Jiao Tong University Chest Hospital 2024 (106)	Clinical data + genetic data	328 Cases	multimodal	Clinical treatment data Genomic data	Multimodal prediction modet	0.798 (NR)
Sichuan University 2024 (107)	Clinical data +CT	24,107 Cases	multimodal	Pathological morphological characteristics Image feature	MMO	0.887 (95% CI: 0.867-0.909)
Beijing University of Aeronautics and Astronautics 2022 (95)	Multicenter CT + gene	18232 Cases	multimodal	Imaging, pathological and clinical features	FAIS	0.748-08 13(95% CI:0.732 - 0.853)
M.d. Anderson Cancer Center 2023 (131)	Pathological data + CT + clinical data	976 Cases	multimodal	Imaging, pathological and clinical features	Deep-CT model Benchmark model	0.75 (NR)
University of Science and Technology of China 2022 (132)	CT + clinical data	570 Cases	multimodal	Metabolic characteristics + imaging features	ESBP	0.754-0.804 (NR)
West China Medical College, Sichuan University 2022 (86)	CT + genetic data + clinical	3816 Cases	multimodal	Imaging, pathological and clinical features	Multimodal prediction modet	0.842 (95% CI, 0.825-0.855)

Analysis of these studies reveals that LLMs excel in NSCLC EGFR mutation prediction by efficiently utilizing unstructured textual data (e.g., pathology reports, clinical notes), addressing the underutilization of such data in traditional approaches. Furthermore, LLMs demonstrate superior contextual understanding, enabling robust cross-modal feature alignment and fusion across imaging, text, and genomic data. Their capacity for temporal data modeling allows tracking of dynamic EGFR mutation changes during treatment, while attention mechanisms and feature contribution analyses provide biologically interpretable insights. However, despite their promise, challenges persist in medical applications of LLMs. Key limitations include the reliance on massive annotated datasets, which are scarce in medicine; high computational demands for training and inference, hindering deployment in resource-limited settings; suboptimal domain-specific terminology comprehension by general-purpose LLMs; and privacy risks during multi-center data sharing and model training.

As artificial intelligence advances in NSCLC mutation prediction, LLMs are driving dual evolutionary pathways: technological paradigm restructuring and clinical value enhancement. Current trends indicate future LLM development will focus on synergistic optimization of efficiency, dynamism, security, and interpretability, bridging the gap from algorithmic validation to clinical implementation. On one front, lightweight architectures (e.g., Med-GPT) leveraging domain-specific knowledge distillation—via parameter pruning and attention mechanism optimization—are reducing computational costs while enhancing analysis of radiomic features (e.g., CT texture heterogeneity) and temporal clinical data (e.g., treatment response dynamics), offering accessible molecular subtyping tools for primary care settings. Concurrently, multimodal dynamic modeling, integrating time-series Transformer architectures with liquid biopsy data (e.g., ctDNA mutation burden), enables construction of spatial-temporal models of EGFR mutation evolution. These models capture dynamic correlations between tumor heterogeneity (e.g., lung nodule volume growth rates) and molecular biomarkers, predicting tyrosine kinase inhibitor (TKI) resistance trajectories. Privacy-preserving technologies, such as federated learning frameworks (FedLLM) with homomorphic encryption and differential privacy, are overcoming multi-center data silos, enabling collaborative training on cross-institutional pathology and genomic datasets. Notably, enhanced interpretability methods (e.g., medical-specific SHAP analysis) are quantifying associations between radiomic features (e.g., ground-glass nodule CT value distributions) and EGFR mutation subtypes (e.g., L858R or exon 19 deletions), building trust between AI predictions and clinical decision-making. This technological evolution not only addresses disparities in genetic testing resource allocation but also promises seamless integration with PACS systems, bridging radiological diagnosis to molecular pathology inference and advancing precision medicine toward preemptive intervention and dynamic monitoring.

## 6 Future prospects and challenges

### 6.1 Data sources and standardization

The current performance bottlenecks of artificial intelligence (AI) models in predicting EGFR mutations in non-small cell lung cancer (NSCLC) primarily stem from fragmented data ecosystems and a lack of standardization. Although multimodal data integration—including CT imaging, histopathology slides, liquid biopsies, and clinical narratives—has emerged as the mainstream paradigm, significant heterogeneity persists in data acquisition protocols. For instance, in medical imaging, variations in CT scanner parameters (e.g., kVp, slice thickness, and reconstruction algorithms) across manufacturers (e.g., Siemens, GE Healthcare, Philips) induce texture feature distribution shifts (domain shift). Studies demonstrate that increasing slice thickness from 1 mm to 5 mm elevates radiomic feature variability by 27%. In genomic profiling, differences in sensitivity between next-generation sequencing (NGS, 0.1%) and digital PCR (1%) introduce substantial labeling noise for low-abundance mutations (e.g., EGFR T790M) (119, 120).

Future efforts must prioritize the development of cross-modal data standardization frameworks. For example, domain adaptation techniques using generative adversarial networks (CycleGAN) could harmonize CT images across scanner vendors, while ISO/IEC 20547-compliant biomedical data lake architectures may enable dynamic alignment and version control of multicenter data.

In data processing, conventional manual annotations (e.g., tumor ROI delineation) suffer from subjectivity and poor reproducibility. Recent advancements propose “hybrid annotation” strategies that integrate expert annotations (following RECIST 1.1 criteria) with weakly supervised learning (e.g., text-image alignment using pathology reports) to extract cross-modality consistent features via contrastive learning. For small-sample mutation subtypes (e.g., EGFR exon 20 insertion mutations, <10% of EGFR mutations), diffusion models (DMs) show promise in synthetic data generation. Experiments reveal that synthetic CT images generated via Stable Diffusion architectures improve model AUC for rare mutations by 12% (121–123).

Furthermore, breakthroughs in federated learning (FL), such as the FedMA algorithm (an enhanced variant of FedAvg), will facilitate cross-institutional collaboration. These frameworks enable distributed model training on global multicenter datasets (e.g., the NSCLC-Radiomics-Genomics Consortium) while ensuring compliance with patient privacy regulations (GDPR/HIPAA).

Cross-population generalisability remains the principal bottleneck. More than 60% of published cohorts originate from single-centre East-Asian datasets, while African-American and Hispanic patients are virtually absent. Scanner-parameter variability is seldom quantified; for example, increasing slice thickness from 1 mm to 5 mm can alter radiomic features by up to 27%. Although the multinational, nine-centre FAIS dataset demonstrates that broader sampling is feasible, such resources remain exceptional. Future work should employ prospective PROBE designs that cover multiple ethnic groups and acquisition protocols, and integrate domain-adaptation or federated-learning strategies to improve out-of-domain performance.

### 6.2 Model interpretability and repeatability

The limited interpretability of artificial intelligence (AI) models has become a critical barrier to their clinical adoption, despite their expanding applications in medicine. While existing methods (e.g., Grad-CAM, LIME) can visualize critical imaging regions (e.g., ground-glass opacity contributing to EGFR mutation predictions), they fail to provide biologically meaningful explanations of molecular mechanisms. Recent research trends focus on cross-modal causal inference to bridge this gap (124). For example, integrating radiomic features with spatial transcriptomic atlases of the tumor microenvironment (TME): spatial transcriptomic sequencing (10x Visium) generates gene expression matrices of tumor regions, and graph attention networks (GATs) establish quantitative models linking CT imaging texture features (e.g., gray-level co-occurrence matrix entropy) to EGFR signaling pathway activation (e.g., PI3K-Akt-mTOR). Such studies have demonstrated that peritumoral vascular tortuosity on CT scans (quantified by fractal dimension) strongly correlates with VEGF overexpression (r=0.73, p<0.001), offering mechanistic insights into imaging-based predictions of EGFR-TKI resistance (125).

For dynamic interpretability, Transformer-based time-series models (e.g., TimeSformer) enable tracking of EGFR mutation evolution during treatment. By integrating serial CT imaging (3-month follow-ups) with ctDNA monitoring data, these models can detect imaging phenotype shifts in EGFR L858R mutations (e.g., reduced ground-glass opacity with increased solid components) and predict the risk of T790M resistance mutations (HR=2.34, 95% CI 1.87–2.93).

Additionally, knowledge graphs enhance model transparency by embedding clinical guidelines (e.g., NCCN), databases (e.g., OncoKB), and imaging features into unified graph structures. This approach renders model decision pathways traceable to evidence-based clinical linkages, such as associations between specific imaging patterns and EGFR-TKI response rates (126, 127).

Our pooled analysis shows that, among the 59 eligible studies, only 11 prediction models (18.6%) have made their source code or executable software publicly available, and none of these releases include the corresponding trained weights or underlying datasets. Furthermore, just one study has employed a prospective, PROBE-style multicentre design and is prospectively registered on ClinicalTrials.gov. The scarcity of shared code and weights makes it impossible to quantify the net clinical benefit, potential risks, and reproducibility of the vast majority of proposed models.

### 6.3 Clinical translation trends

The clinical translation of AI technologies continues to face critical challenges (128), including:

1. Technical Validation Frameworks: Most current studies rely on retrospective single-center data. Future efforts must prioritize prospective multicenter validation platforms (e.g., NCT04253679 trial) employing a Prospective Randomized Open Blinded Endpoint (PROBE) design to evaluate AI models’ impact on clinical endpoints. For example, comparing the detection rate differences (targeting ≥15% improvement) between AI-guided biopsy localization (targeting regions with high predicted mutation probabilities) and conventional random biopsy.
2. Workflow Integration: Developing embedded AI diagnostic systems that seamlessly interface with hospital Picture Archiving and Communication Systems (PACS). The Philips IntelliSpace AI platform has demonstrated real-time CT image analysis capabilities (latency <3 seconds), but its EGFR mutation prediction module still requires FDA’s Center for Devices and Radiological Health (CDRH) certification. A key breakthrough lies in constructing lightweight models (e.g., MobileNetV3-enhanced architectures) that maintain AUC >0.85 while compressing parameter counts to <5M, enabling deployment on edge computing devices (e.g., CT scanner-integrated GPUs).

Future clinical applications of AI will likely follow two trajectories (126, 129, 130):

▪ Dynamic Precision Monitoring: AI-driven “digital twin” models simulating tumor evolution. For instance, generating virtual clones based on baseline CT imaging and genomic data to predict spatiotemporal changes in EGFR mutation abundance under different therapeutic strategies (e.g., osimertinib vs. chemotherapy), thereby guiding personalized treatment planning.
▪ Health Economics Optimization: Markov decision models quantifying AI’s cost-effectiveness ratios. Preliminary studies indicate that AI-guided EGFR mutation screening reduces genomic testing costs by 38% (via minimizing unnecessary NGS assays) while improving targetable population identification rates by 22%.

Ethical and regulatory challenges remain critical. Robust accountability mechanisms must be established to address diagnostic errors (e.g., false negatives causing treatment delays), clarifying legal liabilities. Furthermore, mitigating model bias is paramount—transfer learning strategies tailored to ethnic groups (Asian vs. Caucasian populations) can reduce AUC disparities in EGFR mutation prediction from 0.12 to 0.04.

## 7 Conclusion

Analysis indicates that radiomics-based machine-learning models, which rely on handcrafted feature engineering, require smaller sample sizes and offer high interpretability—advantages in data-limited settings or when traceable decision paths are needed. However, handcrafted features cannot fully characterise the tumour micro-environment, thus capping predictive performance. Deep-learning models, by contrast, learn end-to-end representations that automatically capture high-dimensional textures and contextual cues, uncovering richer EGFR-associated patterns in heterogeneous images and therefore hold a higher theoretical ceiling. Yet, despite encouraging internal results (pooled AUC ≈ 0.84), the absence of external validation, heightened risk of bias, and variability in scanning protocols and patient demographics continue to impede clinical translation.

To advance these models toward bedside application, we advocate: (i) multicentre, prospective PROBE-style studies supplemented by federated learning to broaden data coverage; (ii) domain-adaptation strategies to mitigate scanner and protocol discrepancies; (iii) routine release of model code and weights, decision-curve and cost–benefit analyses; and (iv) enhanced explainability and seamless integration into clinical workflows. Only through sustained verification across diverse populations and devices—and adherence to STARD-AI and CLAIM reporting standards—can AI-based predictors evolve from research prototypes into reliable tools for precision management of lung cancer.

## Data Availability

The original contributions presented in the study are included in the article/supplementary material. further inquiries can be directed to the corresponding authors.

## References

[1] Siegel RL Miller KD Wagle NS Jemal A . Cancer statistics, 2023. CA A Cancer J Clin. (2023) 73:17–48. doi: 10.3322/caac.21763, PMID: 36633525

[2] Sung H Ferlay J Siegel RL Laversanne M Soerjomataram I Jemal A . Global cancer statistics 2020: GLOBOCAN estimates of incidence and mortality worldwide for 36 cancers in 185 countries. Cancer J Clin. (2021) 71:209–49. doi: 10.3322/caac.21660, PMID: 33538338

[3] Majeed U Manochakian R Zhao Y Lou Y . Targeted therapy in advanced non-small cell lung cancer: current advances and future trends. J Hematol Oncol. (2021) 14:1–20. doi: 10.1186/s13045-021-01121-2, PMID: 34238332 PMC8264982

[4] Reck M Remon J Hellmann MD . First-line immunotherapy for non–small-cell lung cancer. J Clin Oncol. (2022) 40:586–97. doi: 10.1200/JCO.21.01497, PMID: 34985920

[5] Zhang X Zhang Y Zhang G Qiu X Tan W Yin X . Deep learning with radiomics for disease diagnosis and treatment: challenges and potential. Front Oncol. (2022) 12:773840. doi: 10.3389/fonc.2022.773840, PMID: 35251962 PMC8891653

[6] Tomaszewski MR Gillies RJ . The biological meaning of radiomic features. Radiology. (2021) 298:505–16. doi: 10.1148/radiol.2021202553, PMID: 33399513 PMC7924519

[7] Taniguchi K Okami J Kodama K Higashiyama M Kato K . Intratumor heterogeneity of epidermal growth factor receptor mutations in lung cancer and its correlation to the response to gefitinib. Cancer Sci. (2008) 99:929–35. doi: 10.1111/j.1349-7006.2008.00782.x, PMID: 18325048 PMC11158886

[8] Chakrabarty N Mahajan A . Imaging analytics using artificial intelligence in oncology: A comprehensive review. Clin Oncol R Coll Radiol. (2023) 36:498–513. doi: 10.1016/j.clon.2023.09.013, PMID: 37806795

[9] Mahajan A Gurukrishna B Wadhwa S Agarwal U Baid U Talbar S . Deep learning based automated epidermal growth factor receptor and anaplastic lymphoma kinase status prediction of brain metastasis in non-small cell lung cancer. Explor Targeting Anti-Tumor Ther. (2023) 4:657–68. doi: 10.37349/etat, PMID: 37745691 PMC10511818

[10] Wang K Lu X Zhou H Gao Y Zheng J Tong M . Deep learning Radiomics of shear wave elastography significantly improved diagnostic performance for assessing liver fibrosis in chronic hepatitis B: A prospective multicentre study. Gut. (2019) 68:729–41. doi: 10.1136/gutjnl-2018-316204, PMID: 29730602 PMC6580779

[11] Yoon HJ Choi J Kim E Um S-W Kang N Kim W . Deep learning analysis to predict EGFR mutation status in lung adenocarcinoma manifesting as pure ground-glass opacity nodules on CT. Front Oncol. (2022) 12:951575. doi: 10.3389/fonc.2022.951575, PMID: 36119545 PMC9478848

[12] Ting DSW Cheung CY-L Lim G Tan GSW Quang ND Gan A . Development and validation of a deep learning system for diabetic retinopathy and related eye diseases using retinalImages from multiethnic populations with diabetes. JAMA. (2017) 318:2211–23. doi: 10.1001/jama.2017.18152, PMID: 29234807 PMC5820739

[13] Lakhani P Sundaram B . Deep learning at chest radiography: automated classification of pulmonary tuberculosis by using convolutional neural networks. Radiology. (2017) 284:574–82. doi: 10.1148/radiol.2017162326, PMID: 28436741

[14] Atmakuru A Chakraborty S Faust O Salvi M Datta Barua P Molinari F . Deep learning in radiology for lung cancer diagnostics: A systematic review of classification, segmentation, and predictive modeling techniques. Expert Syst With Appl. (2024) 255. doi: 10.1016/j.eswa.2024.124665

[15] Yang D Miao Y Liu C Zhang N Zhang D Guo Q . Advances in artificial intelligence applications in the field of lung cancer. Front Oncol. (2024) 14. doi: 10.3389/fonc.2024.1449068, PMID: 39309740 PMC11412794

[16] Javed R Abbas T Khan AH Daud A Bukhari A Alharbey R . Deep learning for lungs cancer detection: a review. Artif Intell Rev. (2024) 57. doi: 10.1007/s10462-024-10807-1

[17] Ferro A Bottosso M Dieci MV Scagliori E Miglietta F Aldegheri V . Clinical applications of radiomics and deep learning in breast and lung cancer: A narrative literature review on current evidence and future perspectives. Crit Rev Oncol Hematol. (2024) 203:104479. doi: 10.1016/j.critrevonc.2024.104479, PMID: 39151838

[18] Quanyang W Yao H Sicong W Linlin Q Zewei Z Donghui H . Artificial intelligence in lung cancer screening: Detection, classification, prediction, and prognosis. Cancer Med. (2024) 13:e7140. doi: 10.1002/cam4.7140, PMID: 38581113 PMC10997848

[19] Hephzibah R Anandharaj HC Kowsalya G Jayanthi R Chandy DA . Review on deep learning methodologies in medical image restoration and segmentation. Curr Med Imaging. (2023) 19:844–54. doi: 10.2174/1573405618666220407112825, PMID: 35392788

[20] Yao W Bai J Liao W Chen Y Liu M Xie Y . From CNN to transformer: A review of medical image segmentation models. J Imaging Inform Med. (2024) 37:1529–47. doi: 10.1007/s10278-024-00981-7, PMID: 38438696 PMC11300773

[21] Xu J Bian Q Li X Zhang A Ke Y Qiao M . Contrastive graph pooling for explainable classification of brain networks. IEEE Trans Med Imaging. (2024) 43:3292–305. doi: 10.1109/TMI.2024.3392988, PMID: 38656865

[22] Ahmed S Jinchao F Ferzund J Ali MU Yaqub M Manan MA . GraFMRI: A graph-based fusion framework for robust multi-modal MRI reconstruction. Magn Reson Imaging. (2025) 116:110279. doi: 10.1016/j.mri.2024.110279, PMID: 39561859

[23] Lim ZW Pushpanathan K Yew SME Lai Y Sun CH Lam JSH . Benchmarking large language models’ performances for myopia care: a comparative analysis of ChatGPT-3.5, ChatGPT-4.0, and Google Bard. EBioMedicine. (2023) 95:104770. doi: 10.1016/j.ebiom.2023.104770, PMID: 37625267 PMC10470220

[24] Shi R Liu S Xu X Ye Z Yang J Le Q . Benchmarking four large language models’ performance of addressing Chinese patients’ inquiries about dry eye disease: A two-phase study. Heliyon. (2024) 10:e34391. doi: 10.1016/j.heliyon.2024.e34391, PMID: 39113991 PMC11305187

[25] Ong JCL Seng BJJ Law JZF Low LL Kwa ALH Giacomini KM . Artificial intelligence, ChatGPT, and other large language models for social determinants of health: Current state and future directions. Cell Rep Med. (2024) 5:101356. doi: 10.1016/j.xcrm.2023.101356, PMID: 38232690 PMC10829781

[26] Matsuo H Nishio M Matsunaga T Fujimoto K Murakami T . Exploring multilingual large language models for enhanced TNM classification of radiology report in lung cancer staging. Cancers (Basel). (2024) 16:3621. doi: 10.3390/cancers16213621, PMID: 39518061 PMC11544964

[27] Tozuka R Johno H Amakawa A Sato J Muto M Seki S . Application of NotebookLM, a large language model with retrieval-augmented generation, for lung cancer staging. Jpn J Radiol. (2024) 43:706–712. doi: 10.1007/s11604-024-01705-1, PMID: 39585559

[28] Wang CK Ke CR Huang MS Chong IW Yang YH Tseng VS . Using large language models for efficient cancer registry coding in the real hospital setting: A feasibility study. Pac Symp Biocomput. (2025) 30:121–37. doi: 10.1142/9789819807024_0010, PMID: 39670366

[29] Megyesfalvi Z Gay CM Popper H Pirker R Ostoros G Heeke S . Clinical insights into small cell lung cancer: Tumor heterogeneity, diagnosis, therapy, and future directions. CA Cancer J Clin. (2023) 73:620–52. doi: 10.3322/caac.21785, PMID: 37329269

[30] Ferber D Wölflein G Wiest IC Ligero M Sainath S Ghaffari Laleh N . In-context learning enables multimodal large language models to classify cancer pathology images. Nat Commun. (2024) 15:10104. doi: 10.1038/s41467-024-51465-9, PMID: 39572531 PMC11582649

[31] Xiao H Zhou F Liu X Liu T Li Z Liu X . A comprehensive survey of large language models and multimodal large language models in medicine. Inf Fusion. (2024). doi: 10.2139/ssrn.5031720

[32] Lei L Xiaoyan Y Junchi L Shen Y Wang J Wei P . A survey on medical large language models: technology, application, trustworthiness, and future directions. arXiv preprint arXiv:2406.03712. (2024) 14. doi: 10.48550/arXiv.2406.03712

[33] Shmatko A Ghafari Laleh N Gerstung M Kather JN . Artificial intelligence in histopathology: enhancing cancer research and clinical oncology. Nat Cancer. (2022) 3:1026–38. doi: 10.1038/s43018-022-00436-4, PMID: 36138135

[34] Unger M Kather JN . A systematic analysis of deep learning in genomics and histopathology for precision oncology. BMC Med Genomics. (2024) 17:48. doi: 10.1186/s12920-024-01796-9, PMID: 38317154 PMC10845449

[35] Echle A Rindtorff NT Brinker TJ Luedde T Pearson AT Kather JN . Deep learning in cancer pathology: a new generation of clinical biomarkers. Br J Cancer. (2021) 124:686–96. doi: 10.1038/s41416-020-01122-x, PMID: 33204028 PMC7884739

[36] Jiang X Hoffmeister M Brenner H Muti HS Yuan T Foersch S . End-to-end prognostication in colorectal cancer by deep learning: a retrospective, multicentre study. Lancet Digit Health. (2024) 6:e33–e43. doi: 10.1016/S2589-7500(23)00208-X, PMID: 38123254

[37] Khader F Müller-Franzes G Wang T Han T Tayebi Arasteh S Haarburger C . Multimodal deep learning for integrating chest radiographs and clinical parameters: a case for transformers. Radiology. (2023) 309:e230806. doi: 10.1148/radiol.230806, PMID: 37787671

[38] Yu AC Mohajer B Eng J . External validation of deep learning algorithms for radiologic diagnosis: a systematic review. Radiol Artif Intell. (2022) 4:e210064. doi: 10.1148/ryai.210064, PMID: 35652114 PMC9152694

[39] Page MJ McKenzie JE Bossuyt PM Boutron I Hoffmann TC Mulrow CD . The PRISMA 2020 statement: An updated guideline for reporting systematic reviews. BMJ. (2021) 372:n71. doi: 10.1016/j.ijsu.2021.105906, PMID: 33782057 PMC8005924

[40] Page MJ Moher D Bossuyt PM Boutron I Hoffmann TC Mulrow CD . PRISMA 2020 explanation and elaboration: Updated guidance and exemplars for reporting systematic reviews. BMJ. (2021) 372:n160. doi: 10.1136/bmj.n160, PMID: 33781993 PMC8005925

[41] Mayerhoefer ME Materka A Langs G Haggstrom I Szczypinski P Gibbs P . Introduction to radiomics. JNuclMed. (2020) 61:488–95. doi: 10.2967/jnumed.118.222893, PMID: 32060219 PMC9374044

[42] Ranstam J Cook JA . Lasso regression. Br J Surg. (2018) 105:1348. doi: 10.1002/bjs.10895

[43] Zhou JY Zheng J Yu ZF Xiao WB Zhao J Sun K . Comparative analysis of clinicoradiologic characteristics of lung adenocarcinomas with alk rearrangements or egfr mutations. Eur Radiol. (2015) 25:1257–66. doi: 10.1007/s00330-014-3516-z, PMID: 25577516

[44] Rizzo S Petrella F Buscarino V De Maria F Raimondi S Barberis M . Ct radiogenomic characterization of egfr, K-ras, and alk mutations in non-small cell lung cancer. Eur Radiol. (2016) 26:32–42. doi: 10.1007/s00330-015-3814-0, PMID: 25956936

[45] Yin X Liao H Yun H Lin N Li S Xiang Y . Artificial intelligence-based prediction of clinical outcome in immunotherapy and targeted therapy of lung cancer. Semin Cancer Biol. (2022) 86:146–59. doi: 10.1016/j.semcancer.2022.08.002, PMID: 35963564

[46] Li S Li Y Zhao M Wang P Xin J . Combination of (18)F-fluorodeoxyglucose pet/ct radiomics and clinical features for predicting epidermal growth factor receptor mutations in lung adenocarcinoma. Korean J Radiol. (2022) 23:921–30. doi: 10.3348/kjr.2022.0295, PMID: 36047542 PMC9434738

[47] Zhao W Yang J Ni B Bi D Sun Y Xu M . Toward automatic prediction of egfr mutation status in pulmonary adenocarcinoma with 3d deep learning. Cancer Med. (2019) 8:3532–43. doi: 10.1002/cam4.2019.8.issue-7, PMID: 31074592 PMC6601587

[48] Huang W Wang J Wang H Zhang Y Zhao F Li K . Pet/ct based egfr mutation status classification of nsclc using deep learning features and radiomics features. Front Pharmacol. (2022) 13:898529. doi: 10.3389/fphar.2022.898529, PMID: 35571081 PMC9092283

[49] Zhang M Bao Y Rui W Shangguan C Liu J Xu J . Performance of (18)F-fdg pet/ct radiomics for predicting egfr mutation status in patients with non-small cell lung cancer. Front Oncol. (2020) 10:568857. doi: 10.3389/fonc.2020.568857, PMID: 33134170 PMC7578399

[50] Nair JKR Saeed UA McDougall CC Sabri A Kovacina B Raidu BVS . Radiogenomic models using machine learning techniques to predict egfr mutations in non-small cell lung cancer. Can Assoc Radiol J. (2021) 72:109–19. doi: 10.1177/0846537119899526, PMID: 32063026

[51] Touvron H Lavril Thibaut Izacard Gautier Martinet Xavier Lachaux Marie-Anne Lacroix Timothee . LLaMA: open and efficient foundation language models. arXiv. (2023). doi: 10.48550/arXiv.2302.13971

[52] Foersch S Glasner C Woerl AC Eckstein M Wagner DC Schulz S . Multistain deep learning for prediction of prognosis and therapy response in colorectal cancer. Nat Med. (2023) 29:430–9. doi: 10.1038/s41591-022-02134-1, PMID: 36624314

[53] Boehm KM Khosravi P Vanguri R Gao J Shah SP . Harnessing multimodal data integration to advance precision oncology. Nat Rev Cancer. (2022) 22:114–26. doi: 10.1038/s41568-021-00408-3, PMID: 34663944 PMC8810682

[54] Delzell D Magnuson S Peter T Smith M Smith BJ . Machine learning and feature selection methods for disease classification with application to lung cancer screening image data. Front Oncol. (2019) 9:1393. doi: 10.3389/fonc.2019.01393, PMID: 31921650 PMC6917601

[55] Pineda AL Ogoe HA Balasubramanian JB Escareño CR Visweswaran S Herman JG . ‘‘On predict-ing lung cancer subtypes using ‘omic’ data from tumor and tumor-adjacent histologically-normal tissue,’’. BMCCancer. (2016) 16:184. doi: 10.1186/s12885-016-2223-3, PMID: 26944944 PMC4778315

[56] Chang C Zhou S Yu H Zhao W Ge Y Duan S . A clinically practical radiomics-clinical combined model based on PET/CT data and nomogram predicts EGFR mutation in lung adenocarcinoma. Eur Radiol. (2021) 31:6259–68. doi: 10.1007/s00330-020-07676-x, PMID: 33544167

[57] Huang L Cao Y Zhou F Li J Ren J Zhang G . Lung adenocarcinoma: development of nomograms based on PET/CT images for prediction of epidermal growth factor receptor mutation status and subtypes. Nucl Med Commun. (2022) 43:310–22. doi: 10.1097/MNM.0000000000001519, PMID: 34954763

[58] Hong D Xu K Zhang L Wan X Guo Y . Radiomics signature as a predictive factor for EGFR mutations in advanced lung adenocarcinoma. Front Oncol. (2020) 10:28. doi: 10.3389/fonc.2020.00028, PMID: 32082997 PMC7005234

[59] Jia TY Xiong JF Li XY Yu W Xu ZY Cai XW . Identifying EGFR mutations in lung adenocarcinoma by noninvasive imaging using radiomics features and random forest modeling. Eur Radiol. (2019) 29:4742–50. doi: 10.1007/s00330-019-06024-y, PMID: 30778717

[60] Jiang M Zhang Y Xu J . Assessing EGFR gene mutation status in non-small cell lung cancer with imaging features from PET/CT. Nucl Med Commun. (2019) 40:842–9. doi: 10.1097/MNM.0000000000001043, PMID: 31290849

[61] Jiang X Ren M Shuang X Yang H Shi D Lai Q . Multiparametric MRI-based radiomics approaches for preoperative prediction of EGFR mutation status in spinal bone metastases in patients with lung adenocarcinoma. J Magn Reson Imaging. (2021) 54:497–507. doi: 10.1002/jmri.27579, PMID: 33638577

[62] Mu W Jiang L Zhang J Shi Y Gray JE Tunali I . Non-invasive decision support for NSCLC treatment using PET/CT radiomics. Nat Commun. (2020) 11:5228. doi: 10.1038/s41467-020-19116-x, PMID: 33067442 PMC7567795

[63] Le NQK Kha QH Nguyen VH Chen YC Cheng SJ Chen CY . Machine learning-based radiomics signatures for EGFR and KRAS mutations prediction in non-small-cell lung cancer. Int J Mol Sci. (2021) 22:17. doi: 10.3390/ijms22179254, PMID: 34502160 PMC8431041

[64] Liu G Xu Z Ge Y Jiang B Groen H Vliegenthart R . 3D radiomics predicts EGFR mutation, exon-19 deletion and exon-21 L858R mutation in lung adenocarcinoma. Transl Lung Cancer Res. (2020) 9:1212–24. doi: 10.21037/tlcr-20-122, PMID: 32953499 PMC7481623

[65] Lu L Sun SH Yang H E L Guo P Schwartz LH . Radiomics prediction of EGFR status in lung cancer – our experience in using multiple feature extractors and the cancer imaging archive data. Tomography. (2020) 6:223–30. doi: 10.18383/j.tom.2020.00017, PMID: 32548300 PMC7289249

[66] Ninomiya K Arimura H Chan WY Tanaka K Mizuno S Muhammad Gowdh NF . Robust radiogenomics approach to the identification of EGFR mutations among patients with NSCLC from three different countries using topologically invariant Betti numbers. PloS One. (2021) 16:e0244354. doi: 10.1371/journal.pone.0244354, PMID: 33428651 PMC7799813

[67] Rossi G Barabino E Fedeli A Ficarra G Coco S Russo A . Radiomic detection of EGFR mutations in NSCLC. Cancer Res. (2021) 81:724–31. doi: 10.1158/0008-5472.CAN-20-0999, PMID: 33148663

[68] Li S Ding C Zhang H Song J Wu L . Radiomics for the prediction of EGFR mutation subtypes in non-small cell lung cancer. Med Phys. (2019) 46:4545–52. doi: 10.1002/mp.v46.10 31376283

[69] Tu W Sun G Fan L Wang Y Xia Y Guan Y . Radiomics signature: a potential and incremental predictor for EGFR mutation status in NSCLC patients, comparison with CT morphology. Lung Cancer. (2019) 132:28–35. doi: 10.1016/j.lungcan.2019.03.025, PMID: 31097090

[70] Weng Q Hui J Wang H Lan C Huang J Zhao C . Radiomic feature-based nomogram: a novel technique to predict EGFR-activating mutations for EGFR tyrosin kinase inhibitor therapy. Front Oncol. (2021) 11:590937. doi: 10.3389/fonc.2021.590937, PMID: 34422624 PMC8377542

[71] Ruan D Fang J Teng X . Efficient 18f-fluorodeoxyglucose positron emission tomography/computed tomography-based machine learning model for predicting epidermal growth factor receptor mutations in non-small cell lung cancer. Q J Nucl Med Mol Imaging. (2022) 68:70–83. doi: 10.23736/S1824-4785.22.03441-0, PMID: 35420272

[72] Shiri I Amini M Nazari M Hajianfar G Haddadi Avval A Abdollahi H . Impact offeature harmonization on radiogenomics analysis: prediction of egfr and kras mutations from non-small cell lung cancer pet/ct images. Comput Biol Med. (2022) 142:105230. doi: 10.1016/j.compbiomed.2022.105230, PMID: 35051856

[73] Naimi AI Balzer LB . Stacked generalization: an introduction to super learning. Eur J Epidemiol. (2018) 33:459–64. doi: 10.1007/s10654-018-0390-z, PMID: 29637384 PMC6089257

[74] Papadimitroulas P Brocki L Christopher Chung N MarChadour W Vermet F Gaubert L . Artificial intelligence: deep learning in oncological radiomics and challenges of interpretability and data harmonization. Physica Med PM. (2021) 83:108–21. doi: 10.1016/j.ejmp.2021.03.009, PMID: 33765601

[75] Mahajan A Chakrabarty N Majithia J Ahuja A Agarwal U Suryavanshi S . Multisystem imaging recommendations/guidelines: in the pursuit of precision oncology. Indian J Med Paediatr Oncol. (2023) 44:2–25. doi: 10.1055/s-0043-1761266

[76] Xiao Z Cai H Wang Y Cui R Huo L Lee EY . Deep learning for predicting epidermal growth factor receptor mutations of non-small cell lung cancer on pet/ct images. Quant Imaging Med Surg. (2023) 13:1286–99. doi: 10.21037/qims-22-760, PMID: 36915325 PMC10006109

[77] Le VH Minh TNT Kha QH Le NQK . Deep learning radiomics for survival prediction in non-small-cell lung cancer patients from CT images. J Med Syst. (2025)49:22. doi: 10.1007/s10916-025-02156-5, PMID: 39930275

[78] Le NQK . Hematoma expansion prediction: still navigating the intersection of deep learning and radiomics. Eur Radiol. (2024) 34:2905–7. doi: 10.1007/s00330-024-10586-x, PMID: 38252277

[79] Nguyen HS Ho DKN Nguyen NN Tran HM Tam KW Le NQK . Predicting EGFR mutation status in non-small cell lung cancer using artificial intelligence: A systematic review and meta-analysis. Acad Radiol. (2024) 31:660–83. doi: 10.1016/j.acra.2023.03.040, PMID: 37120403

[80] Wang S Shi J Ye Z Dong D Yu D Zhou M . Predicting EGFR mutation status in lung adenocarcinoma on computed tomography image using deep learning. Eur Respir J. (2019) 53. doi: 10.1183/13993003.00986-2018, PMID: 30635290 PMC6437603

[81] Wang S Yu H Gan Y Wu Z Li E Li X . Mining whole-lung information by artificial intelligence for predicting EGFR genotype and targeted therapy response in lung cancer: a multicohort study. Lancet Digit Health. (2022) 4:e309–e319. doi: 10.1016/S2589-7500(22)00024-3, PMID: 35341713

[82] Gui D Song Q Song B Li H Wang M Min X . AIR-Net: A novel multi-task learning method with auxiliary image reconstruction for predicting EGFR mutation status on CT images of NSCLC patients. Comput Biol Med. (2022) 141:105157. doi: 10.1016/j.compbiomed.2021.105157, PMID: 34953355

[83] Wang C Ma J Shao J Zhang S Li J Yan J . Non-invasive measurement using deep learning algorithm based on multi-source features fusion to predict PD-L1 expression and survival in NSCLC. Front Immunol. (2022) 13:828560. doi: 10.3389/fimmu.2022.828560, PMID: 35464416 PMC9022118

[84] Song Z Liu T Shi L Yu Z Shen Q Xu M . The deep learning model combining CT image and clinicopathological information for predicting ALK fusion status and response to ALK-TKI therapy in non-small cell lung cancer patients. Eur J Nucl Med Mol Imaging. (2021) 48:361–71. doi: 10.1007/s00259-020-04986-6, PMID: 32794105

[85] Tian P He B Mu W Liu K Liu L Zeng H . Assessing PD-L1 expression in non-small cell lung cancer and predicting responses to immune checkpoint inhibitors using deep learning on computed tomography images. Theranostics. (2021) 11:2098–107. doi: 10.7150/thno.48027, PMID: 33500713 PMC7797686

[86] Wang C Ma J Shao J Zhang S Liu Z Yu Y . Predicting EGFR and PD-L1 status in NSCLC patients using multitask AI system based on CT images. Front Immunol. (2022) 13:813072. doi: 10.3389/fimmu.2022.813072, PMID: 35250988 PMC8895233

[87] Zhang T Xu Z Liu G Jiang B de Bock GH Groen HJM . Simultaneous identification of EGFR,KRAS,ERBB2, and TP53 mutations in patients with non-small cell lung cancer by machine learning-derived three-dimensional radiomics. Cancers (Basel). (2021) 13. doi: 10.3390/cancers13081814, PMID: 33920322 PMC8070114

[88] Zhao Y Xiong S Ren Q Wang J Li M Yang L . Deep learning using histological images for gene mutation prediction in lung cancer: a multicentre retrospective study. Lancet Oncol. (2025) 26:136–46. doi: 10.1016/S1470-2045(24)00599-0, PMID: 39653054

[89] Song J Ding C Huang Q Luo T Xu X Chen Z . Deep learning predicts epidermal growth factor receptor mutation subtypes in lung adenocarcinoma. Med Phys. (2021) 48:7891–9. doi: 10.1002/mp.15307, PMID: 34669994

[90] Zhang X Zhang G Qiu X Yin J Tan W Yin X . Exploring non-invasive precision treatment in non-small cell lung cancer patients through deep learning radiomics across imaging features and molecular phenotypes. biomark Res. (2024)12:12. doi: 10.1186/s40364-024-00561-5, PMID: 38273398 PMC10809593

[91] Dong Y Hou L Yang W Han J Wang J Qiang Y . Multi-channel multi-task deep learning for predicting EGFR and KRAS mutations of non-small cell lung cancer on CT images. Quant Imaging Med Surg. (2021) 11:2354–75. doi: 10.21037/qims-20-600, PMID: 34079707 PMC8107307

[92] Kim S Lim JH Kim CH Roh J You S Choi JS . Deep learning-radiomics integrated noninvasive detection of epidermal growth factor receptor mutations in non-small cell lung cancer patients. Sci Rep. (2024) 14:922. doi: 10.1038/s41598-024-51630-6, PMID: 38195717 PMC10776765

[93] Wang C Xu X Shao J Zhou K Zhao K He Y . Deep learning to predict EGFR mutation and PD-L1 expression status in non-small-cell lung cancer on computed tomography images. J Oncol. (2021) 2021:5499385. doi: 10.1155/2021/5499385, PMID: 35003258 PMC8741343

[94] Mahajan A Kania V Agarwal U Ashtekar R Shukla S Patil VM . Deep-learning-based predictive imaging biomarker model for EGFR mutation status in non-small cell lung cancer from CT imaging. Cancers (Basel). (2024) 16:1130. doi: 10.3390/cancers16061130, PMID: 38539465 PMC10968632

[95] Wang S Yu H Gan Y Wu Z Li E Li X . Mining whole-lung information by artificial intelligence for predicting EGFR genotype and targeted therapy response in lung cancer: a multicohort study. Lancet Digit Health. (2022) 4:e309-e319. doi: 10.1016/S2589-7500(22)00024-3, PMID: 35341713

[96] Wu J Meng H Zhou L Wang M Jin S Ji H . Habitat radiomics and deep learning fusion nomogram to predict EGFR mutation status in stage I non-small cell lung cancer: a multicenter study. Sci Rep. (2024) 14:15877. doi: 10.1038/s41598-024-66751-1, PMID: 38982267 PMC11233600

[97] Ashayeri H Sobhi N Pławiak P Pedrammehr S Alizadehsani R Jafarizadeh A . Transfer learning in cancer genetics, mutation detection, gene expression analysis, and syndrome recognition. Cancers (Basel). (2024) 16:2138. doi: 10.3390/cancers16112138, PMID: 38893257 PMC11171544

[98] Lococo F Ghaly G Flamini S Campanella A Chiappetta M Bria E . Artificial intelligence applications in personalizing lung cancer management: state of the art and future perspectives. J Thorac Dis. (2024) 16:7096–110. doi: 10.21037/jtd-24-244, PMID: 39552872 PMC11565297

[99] Wu G Jochems A Refaee T Ibrahim A Yan C Sanduleanu S . Structural and functional radiomics for lung cancer. Eur J Nucl Med Mol Imaging. (2021) 48:3961–74. doi: 10.1007/s00259-021-05242-1, PMID: 33693966 PMC8484174

[100] Zhang J Yao H Lai C Sun X Yang X Li S . A novel multimodal prediction model based on DNA methylation biomarkers and low-dose computed tomography images for identifying early-stage lung cancer. Chin J Cancer Res. (2023) 35:511–25. doi: 10.21147/j.issn.1000-9604.2023.05.08, PMID: 37969955 PMC10643339

[101] Tong S Huang K Xing W Chu Y Nie C Ji L . Unveiling the distinctive variations in multi-omics triggered by TP53 mutation in lung cancer subtypes: An insight from interaction among intratumoral microbiota, tumor microenvironment, and pathology. Comput Biol Chem. (2024) 113:108274. doi: 10.1016/j.compbiolchem.2024.108274, PMID: 39531992

[102] Wu S Shen G Mao J Gao B . CT radiomics in predicting EGFR mutation in non-small cell lung cancer: a single institutional study. Front Oncol. (2020) 10:542957. doi: 10.3389/fonc.2020.542957, PMID: 33117680 PMC7576846

[103] Lu MY Chen TY Williamson DFK Zhao M Shady M Lipkova J . AI-based pathology predicts origins for cancers of unknown primary. Nature. (2021) 594:106–10. doi: 10.1038/s41586-021-03512-4, PMID: 33953404

[104] Jee J Fong C Pichotta K Tran TN Luthra A Waters M . Automated real-world data integration improves cancer outcome prediction. Nature. (2024) 636:728–36. doi: 10.1038/s41586-024-08167-5, PMID: 39506116 PMC11655358

[105] Xiang J Wang X Zhang X Xi Y Eweje F Chen Y . A vision-language foundation model for precision oncology. Nature. (2025) 638 (8051):769–78. doi: 10.1038/s41586-024-08378-w, PMID: 39779851 PMC12295649

[106] Ai X Jia B He Z Zhang J Zhuo M Zhao J . Noninvasive early identification of durable clinical benefit from immune checkpoint inhibition: a prospective multicenter study (NCT04566432). Signal Transduct Target Ther. (2024) 9:350. doi: 10.1038/s41392-024-02060-3, PMID: 39676097 PMC11646999

[107] Shao J Ma J Yu Y Zhang S Wang W Li W . A multimodal integration pipeline for accurate diagnosis, pathogen identification, and prognosis prediction of pulmonary infections. Innovation (Camb). (2024) 5:100648. doi: 10.1016/j.xinn.2024.100648, PMID: 39021525 PMC11253137

[108] Lee JE Park KS Kim YH Song HC Park B Jeong YJ . Lung cancer staging using chest CT and FDG PET/CT free-text reports: comparison among three chatGPT large language models and six human readers of varying experience. AJR Am J Roentgenol. (2024) 223:e2431696. doi: 10.2214/AJR.24.31696, PMID: 39230409

[109] Kim S Jang S Kim B Sunwoo L Kim S Chung JH . Automated pathologic TN classification prediction and rationale generation from lung cancer surgical pathology reports using a large language model fine-tuned with chain-of-thought: algorithm development and validation study. JMIR Med Inform. (2024) 12:e67056. doi: 10.2196/67056, PMID: 39705675 PMC11699504

[110] Park HJ Huh JY Chae G Choi MG . Extraction of clinical data on major pulmonary diseases from unstructured radiologic reports using a large language model. PloS One. (2024) 19:e0314136. doi: 10.1371/journal.pone.0314136, PMID: 39585830 PMC11588275

[111] Yamagishi Y Nakamura Y Hanaoka S Abe O . Large language model approach for zero-shot information extraction and clustering of Japanese radiology reports: algorithm development and validation. JMIR Cancer. (2025) 11:e57275. doi: 10.2196/57275, PMID: 39864093 PMC11867198

[112] Singhal K Azizi S Tu T Mahdavi SS Wei J Chung HW . Large language models encode clinical knowledge. Nature. (2023) 620:172–80. doi: 10.1038/s41586-023-06291-2, PMID: 37438534 PMC10396962

[113] Kefeli J Tatonetti N . Generalizable and automated classification of TNM stage from pathology reports with external validation. medRxiv. (2023) 15:8916. doi: 10.1101/2023.06.26.23291912, PMID: 39414770 PMC11484761

[114] Lu MY Chen B Williamson DFK Chen RJ Zhao M Chow AK . A multimodal generative AI copilot for human pathology. Nature. (2024) 634:466–73. doi: 10.1038/s41586-024-07618-3, PMID: 38866050 PMC11464372

[115] Wang X Zhao J Marostica E Yuan W Jin J Zhang J . A pathology foundation model for cancer diagnosis and prognosis prediction. Nature. (2024) 634:970–8. doi: 10.1038/s41586-024-07894-z, PMID: 39232164 PMC12186853

[116] Liu X Liu H Yang G Jiang Z Cui S Zhang Z . A generalist medical language model for disease diagnosis assistance. Nat Med. (2025) 31:932–42. doi: 10.1038/s41591-024-03416-6., PMID: 39779927

[117] Wu SH Tong WJ Li MD Hu HT Lu XZ Huang ZR . Collaborative enhancement of consistency and accuracy in US diagnosis of thyroid nodules using large language models. Radiology. (2024) 310:e232255. doi: 10.1148/radiol.232255, PMID: 38470237

[118] Song M Wang J Yu Z Wang J Yang L Lu Y . PneumoLLM: Harnessing the power of large language model for pneumoconiosis diagnosis. Med image Anal. (2024) 97:103248. doi: 10.1016/j.media.2024.103248, PMID: 38941859

[119] Zhao W Wu Y Xu Y Sun Y Gao P Tan M . The potential of radiomics nomogram in non- invasively prediction of epidermal growth factor receptor mutation status and subtypes in lung adenocarcinoma. Front Oncol. (2019) 9:1485. doi: 10.3389/fonc.2019.01485, PMID: 31993370 PMC6962353

[120] Zhu Y Guo YB Xu D Zhang J Liu ZG Wu X . A computed tomography (CT)-derived radiomics approach for predicting primary co-mutations involving TP53 and epidermal growth factor receptor (EGFR) in patients with advanced lung adenocarcinomas (LUAD). Ann Transl Med. (2021) 9:545. doi: 10.21037/atm-20-6473, PMID: 33987243 PMC8105857

[121] Acosta JN Falcone GJ Rajpurkar P Topol EJ . Multimodal biomedical AI. Nat Med. (2022) 28:1773–84. doi: 10.1038/s41591-022-01981-2, PMID: 36109635

[122] Warnat-Herresthal S Schultze H Shastry KL Manamohan S Mukherjee S Garg V . Swarm Learning for decentralized and confidential clinical machine learning. Nature. (2021) 594:265–70. doi: 10.1038/s41586-021-03583-3, PMID: 34040261 PMC8189907

[123] Thomasian NM Kamel IR Bai HX . Machine intelligence in non-invasive endocrine cancer diagnostics. Nat Rev Endocrinol. (2022) 18:81–95. doi: 10.1038/s41574-021-00543-9, PMID: 34754064 PMC8576465

[124] London AJ . Artificial intelligence and black-box medical decisions: accuracy versus explainability. Hastings Cent Rep. (2019) 49:15–21. doi: 10.1002/hast.2019.49.issue-1, PMID: 30790315

[125] Zhao T Chiang ZD Morriss JW LaFave LM Murray EM Del Priore I . Spatial genomics enables multi-modal study of clonal heterogeneity in tissues. Nature. (2022) 601:85–91. doi: 10.1038/s41586-021-04217-4, PMID: 34912115 PMC9301586

[126] Pich O Bailey C Watkins TBK Zaccaria S Jamal-Hanjani M Swanton C . The translational challenges of precision oncology. Cancer Cell. (2022) 40:458–78. doi: 10.1016/j.ccell.2022.04.002, PMID: 35487215

[127] Ricci Lara MA Echeveste R Ferrante E . Addressing fairness in artificial intelligence for medical imaging. Nat Commun. (2022) 13:4581. doi: 10.1038/s41467-022-32186-3, PMID: 35933408 PMC9357063

[128] Perez-Lopez R Ghaffari Laleh N Mahmood F Kather JN . A guide to artificial intelligence for cancer researchers. Nat Rev Cancer. (2024) 24:427–41. doi: 10.1038/s41568-024-00694-7, PMID: 38755439

[129] Castelo-Branco L Pellat A Martins-Branco D Valachis A Derksen JWG Suijkerbuijk KPM . ESMO guidance for reporting oncology real-world evidence (GROW). Ann Oncol. (2023) 34:1097–112. doi: 10.1016/j.annonc.2023.10.001, PMID: 37848160

[130] Huang X Rymbekova A Dolgova O Lao O Kuhlwilm M . Harnessing deep learning for population genetic inference. Nat Rev Genet. (2024) 25:61–78. doi: 10.1038/s41576-023-00636-3, PMID: 37666948

[131] Saad MB Hong L Aminu M Vokes NI Chen P Salehjahromi M . Predicting benefit from immune checkpoint inhibitors in patients with non-small-cell lung cancer by CT-based ensemble deep learning: a retrospective study. Lancet Digit Health. (2023) 5:e404-e420. doi: 10.1016/S2589-7500(23)00082-1, PMID: 37268451 PMC10330920

[132] Deng K Wang L Liu Y Li X Hou Q Cao M . A deep learning-based system for survival benefit prediction of tyrosine kinase inhibitors and immune checkpoint inhibitors in stage IV non-small cell lung cancer patients: A multicenter, prognostic study. EClinicalMedicine. (2022) 51:101541. doi: 10.1016/j.eclinm.2022.101541, PMID: 35813093 PMC9256845

